# Phenotypic spectrum and genetics of *PAX2*-related disorder in the Chinese cohort

**DOI:** 10.1186/s12920-021-01102-x

**Published:** 2021-10-25

**Authors:** Xue Yang, Yaqi Li, Ye Fang, Hua Shi, Tianchao Xiang, Jiaojiao Liu, Jialu Liu, Xiaoshan Tang, Xiaoyan Fang, Jing Chen, Yihui Zhai, Qian Shen, Yunli Bi, Yanyan Qian, Bingbing Wu, Huijun Wang, Wenhao Zhou, Duan Ma, Haitao Bai, Jianhua Mao, Lizhi Chen, Xiaowen Wang, Xiaojie Gao, Ruifeng Zhang, Jieqiu Zhuang, Aihua Zhang, Xiaoyun Jiang, Hong Xu, Jia Rao

**Affiliations:** 1 Department of Nephrology, Children’s Hospital of Fudan University, National Pediatric Medical Center of CHINA, 399 Wanyuan Road, Shanghai, China; 2 Shanghai Kidney Development and Pediatric Kidney Disease Research Center, Shanghai, China; 3 Shanghai Key Lab of Birth Defect, Children’s Hospital of Fudan University, Shanghai, 201102 China; 4 Department of Urology, Children’s Hospital of Fudan University, Shanghai, China; 5 Clinical Genetic Center, Children’s Hospital of Fudan University, Shanghai, China; 6 Key Laboratory of Metabolism and Molecular Medicine, Ministry of Education, Department of Biochemistry and Molecular Biology, Institutes of Biomedical Sciences, School of Basic Medical Sciences, Fudan University, Shanghai, China; 7 The First Affiliated Hospital of Xiamen University, Xiamen, China; 8 The Children Hospital of Zhejiang University School of Medicine, Hangzhou, China; 9 Department of Pediatrics, The First Affiliated Hospital, Sun Yat-Sen University, Guangzhou, 510080 China; 10 Wuhan Children’s Hospital, Tongji Medical College, Huazhong University of Science & Technology, Wuhan, China; 11 Shenzhen Children’s Hospital, Shenzheng, China; 12 Xuzhou Children’s Hospital, Xuzhou, China; 13 The Second Affiliated Hospital and Yuying Children’s Hospital of Wenzhou Medical University, Wenzhou, China; 14 Children’s Hospital of Nanjing Medical University, Nanjing, China; 15 State Key Laboratory of Medical Neurobiology, Institutes of Brain Science and School of Basic Medical Science, Fudan University, Shanghai, China

**Keywords:** Congenital anomalies of the kidneys and urinary tract (CAKUT), *PAX2*, Renal coloboma syndrome (RCS), Phenotypic cluster analysis

## Abstract

**Background:**

Pathogenic variants of *PAX2* cause autosomal-dominant *PAX2*-related disorder, which includes variable phenotypes ranging from renal coloboma syndrome (RCS), congenital anomalies of the kidney and urinary tract (CAKUT) to nephrosis. Phenotypic variability makes it difficult to define the phenotypic spectrum associated with genotype.

**Methods:**

We collected the phenotypes in patients enrolled in the China national multicenter registry who were diagnosed with pathogenic variant in *PAX2* and reviewed all published cases with *PAX2*-related disorders. We conducted a phenotype-based cluster analysis by variant types and molecular modeling of the structural impact of missense variants.

**Results:**

Twenty different *PAX2* pathogenic variants were identified in 32 individuals (27 families) with a diagnosis of RCS (9), CAKUT (11) and nephrosis (12) from the Chinese cohort. Individuals with abnormal kidney structure (RCS or CAKUT group) tended to have likely/presumed gene disruptive (LGD) variants (*Fisher test*, *p* < 0.05). A system review of 234 reported cases to date indicated a clear association of RCS to heterozygous loss-of-function *PAX2* variants (LGD variants). Furthermore, we identified a subset of *PAX2* missense variants in DNA-binding domain predicted to affect the protein structure or protein-DNA interaction associated with the phenotype of RCS.

**Conclusion:**

Defining the phenotypic spectrum combined with genotype in *PAX2*-related disorder allows us to predict the pathogenic variants associated with renal and ophthalmological development. It highlighted the approach of structure-based analysis can be applied to diagnostic strategy aiding precise and timely diagnosis.

**Supplementary Information:**

The online version contains supplementary material available at 10.1186/s12920-021-01102-x.

## Background

PAX2 (Paired Box gene 2) is a transcription factor that plays a vital role during early embryonic kidney development, and mediates the development of eyes, ears, and genital tract [1–4]. Heterozygous variants in *PAX2* were first identified in 1995 in patients with renal coloboma syndrome (RCS, also known as “papillorenal syndrome”, MIM #120330), which is a rare autosomal dominant disorder characterized by renal hypodysplasia (RHD) and retinal coloboma (HP:0000480)[5]. *PAX2* heterozygous variants lead to various renal phenotypes across the morphological continuum of congenital anomalies of the kidney and urinary tract (CAKUT) including RHD (HP:0000089, 65%), vesicoureteral reflux (VUR, HP:0000076, 14%), renal cysts (HP:0000107, 8%) and multicystic dysplastic kidneys (HP:0000003, 6%) [6]. Furthermore, phenotypic variability is reported in patients ranging from typical RCS with CAKUT to nephrosis (pathological changes of focal segmental glomerulosclerosis) without renal morphological abnormalities [7–9]. Notably, there is a high degree of phenotypic variability between individuals with *PAX2* variants even in the same pedigree.

**Fig. 1:**
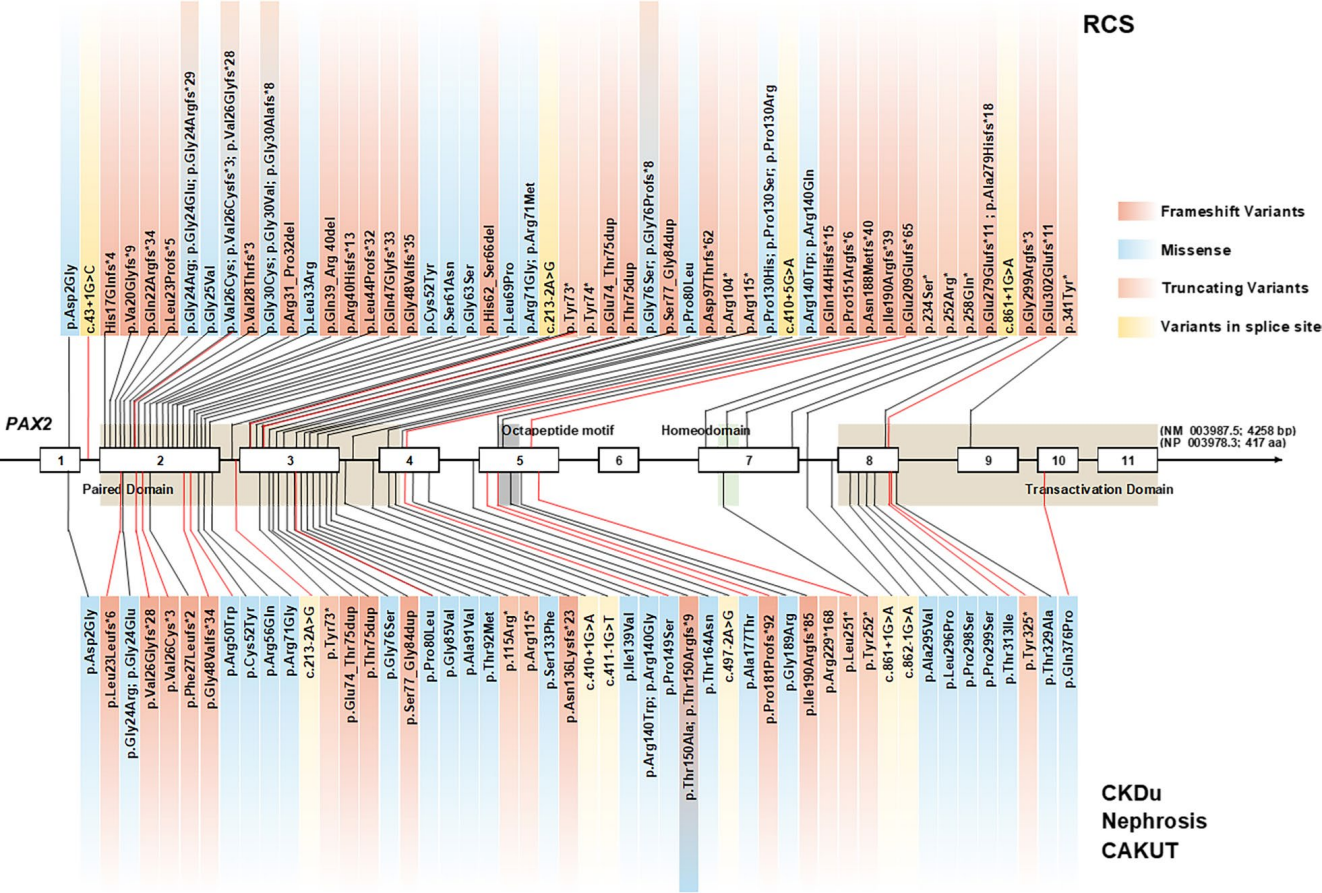
Genetic variants of *PAX2* that associated with kidney disease. The localization and effect on the protein sequence of all reported *PAX2* pathogenic variants (90, labeled with black lines) and pathogenic variants (20, labeled with red lines) identified from the Chinese Children Genetic Kidney Disease Database (CCGKDD) were highlighted. The upper panel showed the variants identified in patients with renal coloboma syndrome (RCS). The lower panel showed the variants identified in patients with isolated CAKUT or nephrosis or CKD of unknown etiology (CKDu). The middle panel showed the exon/intron structure of the human *PAX2* gene. The functional domains are shaded in different colors: N-terminal paired domain (linen) and, Octapeptide motif (grey), homeodomain (olive) and C-terminal Transactivation domain (linen). Frameshift variants (boxed in magnifier), truncating variants (boxed in cantaloupe), splice site variants (boxed in yellow) and missense variants (boxed in blue) were shown, respectively. Residues with two colors represented the variants for different types in the same residual position

Human *PAX2* encodes a multidomain transcription factor characterized by an N-terminal DNA binding paired domain (consists of N-terminal subdomain and C-terminal subdomain, residues 16–142), octapeptide motif (residues 185–192), homeodomain (250–278), and a transactivation domain (279–373) at C-terminus (Fig. 1) [9, 10]. Haploinsufficiency of *PAX2* has been reported among the patients with RCS, CAKUT or focal segmental glomerular sclerosis (FSGS) caused by the likely/presumed gene disruptive (LGD) variants or missense variants that may mimic haploinsufficiency. However, despite many reports of pathogenic variants in the *PAX2* gene, there have been few reports revealing a consistent genotype–phenotype correlation [6, 8, 11].

Here we analyzed *PAX2* pathogenic variants from the Chinese Children Genetic Kidney Disease Database (CCGKDD) which has assembled the largest genetically screened cohort with childhood kidney disease in China to date [12]. And we reviewed all published cases of *PAX2* pathogenic variants to explore the genotypic and phenotypic spectrum of *PAX2*-related disorders. An approach to define the phenotypic spectrum associated with *PAX2* missense variants was established to determine how confidently the clinical phenotype can be predicted by quantifying the pathogenicity of amino acid substitutions.

## Methods

### Study design and participants

Among the individuals with kidney disease consecutively enrolled in the national multicenter registry CCGKDD (www. ccgkdd.com.cn) from 2014 to 2020, patients were recruited with the diagnosis of *PAX2*-related disorder. Enrolled participants were solicited via clinicians from the CCGKDD [12]. Participants were asked to provide information on presenting clinical features, genetic diagnosis (trio-exome sequencing or family based-exome sequencing), and medical management, age at the clinical events (initial presentation, end stage renal disease, ESRD, transplantation), and status (with native renal function, dialysis, transplantation, deceased) at the last follow-up. Patients enrolled the study had been followed up more than 12 months. No identifying information was collected about patients or respondents. The name of the reporting center was collected to allow comparison of entries to avoid duplicates.

*PAX2-*related disorder diagnosis was confirmed based on the clinical phenotype and presence of a genetic mutation through exome sequencing. Clinical phenotypes were stratified by the abnormal findings of urinalysis or radiology. Patients group of RCS (papillorenal syndrome) was defined on the basis of the renal developing deficiency combined with ocular anomalies. Patients without ocular anomalies who was diagnosed with VUR, unilateral or bilateral renal hypodysplasia or multicystic dysplastic kidney were classified as the isolated CAKUT group. Patients presented initially with proteinuria or hematuria were classified as the nephrosis group.

### Genetic analysis

Samples of enrolled individuals were subjected to whole-exome sequencing (WES) of parent–child trios upon obtaining informed consent. The annotation of the WES procedure and its variants has been described in detail previously [12]. The variant interpretation was performed manually by a panel of nephrologists and clinical molecular geneticists [12]. For clinical sequence interpretation, variants were classified according to the American College of Medical Genetics and Genomics (ACMG) guidelines. The Human Gene Mutation Database (HGMD), LOVD (Leiden Open Variation Database) and ClinVar databases were used to search the known disease causing *PAX2* variants. All information on the pathogenic variants and variants of uncertain significance (VUS) of known pathogenic genes can be found from the website of www.ccgkdd.com.cn with a guest account (browse only, not download). Nucleotide and amino acid sequence changes are recorded using the following the National Center for Biotechnology Information RefSeq accession number of *PAX2* (NM_003987 and NP_003978).

### Literature review for *PAX2*-related disorder

We collected reported loci with references from public database: Human Gene Mutation Database (HGMD, http://www.hgmd.cf.ac.uk/ac), Leiden Open Variation Database (LOVD, http://www.lovd.nl/3.0/http://LOVD.nl/GENE), Clinvar database (https://www.ncbi.nlm.nih.gov/clinvar) and literature searching. *PAX2* function disturbance due to chromosome aberrant is excluded. We conducted a literature search of PubMed database with the search terms “*PAX2*”, “renal coloboma syndrome”, “FSGS” and “CAKUT” including systematical review of reported *PAX2* mutations, case report of *PAX2* related disorders and retro/prospective study to investigate prevalence of genes including *PAX2* in diseased population. The search procedure was conducted during the first week of November 2020 (start date: November 2nd, 2020; end date: November 8th, 2020). Systemic analysis was performed on the information of genotype and phenotype. Cases without appropriately described phenotypes of kidney involvement, or cases without pathogenic variants identified were excluded. The phenotypic data were curated from original publications. The clustering analyses and generation of the heat maps were performed using the R packages cluster and gplots, and the function heatmap. Within heatmap, the functions dist and daisy were combined to compute the distance/dissimilarity matrix, respectively.

### Protein structural analysis

The structural analysis of PAX2 was performed using crystallographic structure of PAX5 in complex with DNA (PDB accession 1k78). The global sequence alignment between PAX2 and PAX5 indicated the percent identity of 70.44%. The sequence of the paired domain of PAX5 differs from that of PAX2 by just three residues (97,122 and 123), all relatively far from those affected by the mutations, so that the generation of a homology model was not necessary [9]. The effects of all missense variants were modeled with the program FoldX [13, 14], both using the protein monomer by itself and the protein: DNA complex. The effects of all missense variants were modeled with the program FoldX, both using the protein monomer by itself (PDB accession 6pax) and the protein:DNA complex (PDB accession 1k78). Default FoldX parameters were used, with ten replicates performed per variant. Variants with ΔΔG > 1.6 kcal/mol (using the same threshold described previously) for the protein monomer were classified as “destabilize folding” (D). Variants with monomer ΔΔG ≤ 1.6 kcal/mol, where the ΔΔG for the full complex was > 0.8 kcal/mol, were classified as “perturb protein: DNA interaction” (P) [15]. All other variants were classified as “unknown molecular effect” (U). In total, 12 different computational phenotype predictors [16] were run for all of the pathogenic and putatively benign (gnomAD) variants in the primary PAX2 isoform (Uniprot ID: Q02962-1). The predicted properties of all variants are provided in Additional file 1: Table S2.

### Statistics and analysis

Data were analyzed using Excel. Continuous variables were summarized with median, IQR and categorical data were summarized with proportions. Mann–Whitney test (for continuous variables) and the Fisher exact probability test (for categorical variables) were used to analyze the differences.

## Results

### Clinical characteristics of patients with *PAX2*-related disorder

A total of 32 probands of *PAX2*-related disorder were enrolled in this study of 2256 affected individuals with a wide spectrum of kidney diseases on CCGKDD from 2014 to 2020. Thirty-two patients from 27 families presented initially at a median age of 10 years old (IQR, 5–22) with a female/male ratio being 1:1.3. Patient characteristics were shown in Table 1. Twenty-five probands were initially diagnosed with CAKUT and five of them had concomitant proteinuria or hematuria. Six probands were presented with proteinuria, hematuria and chronic kidney disease (CKD 2–5 stage) with unknown etiology and one with a primary diagnosis of steroid resistant nephrotic syndrome. Two probands were clinically diagnosed of CAKUT at birth through prenatal ultrasound. There were multiple phenotypes of kidney development including renal hypodysplasia (22), vesicoureteral reflux (VUR, 6) cystic kidneys (2) and multicystic dysplasia kidney (1). Renal biopsy was performed in 6 patients providing the histopathological changes of FSGS (3), IgA nephropathy (Hass IV type.1), membranous nephropathy (1) and tubulointerstitial nephropathy (1).

**Table 1:** Clinical features and genotypes of the individuals with *PAX2*-related disorder in the study

Patient ID	Gender	Age at initial presentation	Age progressed to ESRD	Phenotype category	Diagnosis of Kidney disease	renal biopsy	VCUG	Ocular phenotype	non-renal, non-ophthalmological phenotypes	Pathogenic variants (c change, p change, segregation)	Type of pathogenic variants	HGMD	Frequency in gomAD (east Asia);Ref SNP ID	ACMG classfication	Segregation	RefSNP ID
C3	M	8 yrs	Unknown	RCS	Bilateral renal hypodysplasia	IgA nephropathy (Hass IV)	Normal	Bilateral optic disk dysplasia	No abnormal	c.43 + 1G > A (de novo)	Splice site	CS122242	None	P		
C18	F	17 yrs	17 yrs	CAKUT	Bilateral renal hypodysplasia, VUR	NA	VUR	Nystagmus	No abnormal	c.69delC, p.Leu23Leufs*6 (de novo)	Frameshift	CD118561	None	P	De novo	
C1	M	1 yrs	10 yrs	RCS	Bilateral renal hypodysplasia, CKD5	NA	NA	Unilateral optic disc coloboma, choroid anomalies	No abnormal	c.76dupG, p.Val26Glyfs*28 (de novo)	Frameshift	CD992538	None	P	De novo	
C2	M	5 yrs	5 yrs	RCS	Multicyclic dysplasia kidney, CKD 5	NA	NA	Bilateral papillary dysplasia	Bilateral oblique inguinal hernia	c.76dupG, p.Val26Glyfs*28 (de novo)	Frameshift	CI951965	None	P	De novo	
C4	F	1 yrs	6 yrs	CAKUT	Bilateral renal hypodysplasia, CKD 5	NA	Abnormal urinary bladder morphology	NA	Unilateral oblique inguinal hernia	c.76dupG, p.Val26Glyfs*28 (de novo)	Frameshift	CI951965	None	P	De novo	
C7	M	6.8 yrs	Unknown	Nephrosis	Bilateral cystic kidney disease, CKD 4	FSGS	NA	Normal	No abnormal	c.76dupG, p.Val26Glyfs*28 (de novo)	Frameshift	CI951965	None	P	De novo	
C8	M	8.2 yrs	Unknown	Nephrosis	Bilateral cystic kidney disease, CKD 4	NA	NA	Normal	β-thalassemia	c.76dupG, p.Val26Glyfs*28(de novo)	Frameshift	CI951965	None	P	De novo	
C16	F	13 yrs	Unknown	RCS	Bilateral renal hypodysplasia, CKD 4	NA	Bilateral	Unilateral optic disk dysplasia	No abnormal	c.76dupG, p.Val26Glyfs*28 (de novo)	Frameshift	CI951965	None	P	De novo	
919	M	24 yrs	Unknown	Nephrosis	Bilateral renal hypodysplasia, CKD 3	NA	NA	NA	No abnormal	c.76dupG, p.Val26Glyfs*28 (het; sibling, het)	Frameshift	CI951965	None	P	Affected siblings	
920	M	22 yrs	Unknown	Nephrosis	Bilateral renal hypodysplasia, CKD 2	NA	NA	NA	No abnormal	c.76dupG, p.Val26Glyfs*28(het; sibling, het)	Frameshift	CI951965	None	P	Affected siblings	
C12	M	1 d	Unknown	CAKUT	Bilateral renal hypodysplasia	NA	NA	NA	No abnormal	c.81-103delinsC, p.Val28Thrfs*3 (de novo)	Frameshift	NA	None	P	De novo	
C14	M	2 yrs	Unknown	CAKUT	Bilateral renal hypodysplasia, CKD 3	Bilateral renal hypodysplasia	NA	NA	No abnormal	c.143delG, p.Gly48Valfs*34 (de novo)	Frameshift	NA	None	P	De novo	
367	F	23yrs	25 yrs	Nephrosis	CKD5 of unknown etiology	NA	NA	Normal	High-frequency hearing loss	c.148C > T, p.Arg50Trp (het; m,wt; sibling2, het; sibling3,het)	Missense	NA	None;rs759356936	LP	Affected siblings	rs759356936
368	F	34yrs	37yrs	Nephrosis	CKD5 of unknown etiology	NA	NA	Normal	High-frequency hearing loss	c.148C > T, p.Arg50Trp (het; m,wt; sibling1, het; sibling3,het)	Missense	NA	None;rs759356936	LP	Affected siblings	rs759356936
369	F	35yrs	36yrs	Nephrosis	CKD5 of unknown etiology	NA	NA	Normal	High-frequency hearing loss	c.148C > T, p.Arg50Trp (het; m,wt; sibling1, het; sibling2,het)	Missense	NA	None;rs759356936	LP	Affected siblings	rs759356936
C5	F	10 yrs	10 yrs	RCS	Bilateral renal hypodysplasia, bilateral VUR, CKD 5, left double ureters	NA	Bilateral VUR grade II	Bilateral retinal atrophy/dysplasia	Development dysplasia of right hip	c.219C > G, p.Try73* (het; p,het; m,wt)	Nonsense	CM122226	None	P	paternal	
C13	F	Prenatal	Unknown	RCS	Bilateral renal hypodysplasia, CKD 3	NA	NA	Unilateral optic disc coloboma	No abnormal	c.221_226dupAGACCG, p.Glu74_Thr75dup (de novo)	Insertion	CI983182	None	P	De novo	
C29	F	10 yrs	12 yrs	CAKUT	Bilateral renal hypodysplasia, CKD 5	NA	NA	NA	No abnormal	c.221_226dupAGACCG, p.Glu74_Thr75dup (de novo)	Insertion	CI983182	None	LP	De novo	
C31	F	2.8 yrs	6 yrs	CAKUT	Bilateral renal hypodysplasia, CKD 5	NA	Left VUR grade III	Intermittent strabismus	No abnormal	c.685C > T, p.Arg229*168 (NA #)	Nonsense	NA	None	P	NA	
902	M	22yrs	Unknown	CAKUT	Bilateral renal hypodysplasia, CKD 5	NA		NA	No abnormal	c.239C > A p.Pro80Gln (de novo)	Missense	NA	None	LP	De novo	NA
C11	M	14yrs	Unknown	CAKUT	Unilateral renal hypodysplasia, CKD 3	NA	NA	NA	No abnormal	c.445C > T, p.Pro149Ser (de novo)	Missense	NA	5/18464;rs1401507282	LP	De novo	rs1401507282
C15	M	8 yrs	8 yrs	RCS	Bilateral renal hypodysplasia, VUR, CKD 5	NA		Macular pucker	Seizure	c.451delC p.Pro151Argfs*6	Frameshift	NA	None	P	De novo	
C19	M	3 m	Unknown	CAKUT	Unilateral VUR		Left VUR grade III/IV	NA	No abnormal	c.529G > A, p.Ala177Thr (het; p,het; m,wt)	Missense	NA	0/19952/0.000;rs749684940	LP	Paternal	rs749684940
C10	M	11 yrs	11 yrs	CAKUT	Bilateral renal hypodysplasia, CKD 5	NA	NA	Normal	Seizure	c.543delA, p.Pro181Profs*92 (de novo)	Frameshift	NA	None	P	De novo	
C32	M	2.2 yrs	6 yrs	RCS	Bilateral renal hypodysplasia, CKD 5	NA	VUR I	Left optic disk dysplasia	Hernia	c.627delG, p.Glu209Glufs*65 (de novo)	Frameshift	P	None	P	De novo	
C33	F	9 yrs	10 yrs	CAKUT	Bilateral renal hypodysplasia, CKD 5	NA	VUR left I	Normal	No abnormal	c.213-2A > G (de novo)	Splice site	NA	None	P	De novo	
PD 273	F	11 yrs	11 yrs	RCS	Bilateral renal hypodysplasia, CKD 5	NA	Normal	Left optic disk dysplasia	No abnormal	c.836_c.840del AAGTC,p.Glu279Glufs*11 (de novo)	Frameshift	De novo	None	P	De novo	
C17	F	5 yrs	13 yrs	Nephrosis	Bilateral renal hypodysplasia, CKD 5	FSGS	Normal	NA	Unilateral oblique inguinal hernia	c.906C > A, p.Tyr302*(de novo)	Nonsense	NA	ND	P	De novo	
C9	F	14 yrs	/	Nephrosis	CKD 1, SRNS	Membranous nephropathy (stage I-II)	NA	Normal	No abnormal	c.938C > T, p.Pro313Leu (het; p,het; m,wt)	Missense	NA	ND	LP	paternal	NA
198	M	37 yrs	48 yrs	Nephrosis	CKD5 of unknown etiology	NA	NA	NA	No abnormal	c.1127A > C, p.Gln376Pro (het; sibling 1,het; sibling 2, wt; son,het)	Missense	NA	47/19896;rs201021899	LP	Affected sibling	rs201021899
200	M	27 yrs	/	Nephrosis	CKD2 of unknown etiology	TIN	NA	NA	No abnormal	c.1127A > C, p.Gln376Pro (het; p,het; m, wt; paternal aunt,het; paternal uncle, wt)	Missense	NA	47/19896/0.002362;rs201021899	LP	Maternal	rs201021899
201	F	35 yrs	/	Nephrosis	CKD5 of unknown etiology	FSGS	NA	NA	No abnormal	c.1127A > C, p.Gln376Pro(het; sibling 3,het; sibling 2, wt; son,het)	Missense	NA	47/19896/0.002362;rs201021899	LP	Affected sibling	rs201021899

Nucleotide and amino acid sequence changes are reported using the following National Center for Biotechnology Information RefSeq accession numbers (NM_003987 and NP_003978)

CAKUT, congenital anomalies of the kidney and urinary trac; CKD, chronic kidney disease; ESRD, end stage renal disease; FSGS, focal segmental glomerulosclerosis; m, maternal; N.A., not available; p., paternal; RCS, renal coloboma syndrome; RHD, renal hypodysplasia; VUR, vesicoureteral reflux, yrs, years old

Ophthalmological abnormalities were detected in 11 patients, including optic nerve abnormalities (bilateral, 3; unilateral, 5), macular pucker (1), nystagmus (1), intermittent strabismus (1). Of the remaining patients, 8 patients showed normal fundus examination, whereas only normal visual acuity was reported in 13 patients till the last follow up. Non-renal, non-ophthalmological manifestations were revealed in 11 patients including hernia (4), hearing loss (3), seizure (2), β-thalassemia (1), development dysplasia of right hip (1).

### Genetic spectrum of *PAX2*-related disorder

We identified 20 distinct *PAX2* pathogenic variants in 32 individuals in this *PAX2*-related disorder cohort. De novo variants were identified in 19 patients. In the five families with multiple affected individuals, the variant segregated appropriately with the disease. In the remaining one individual, we could not determine the inheritance of the pathogenic variant because the required samples were not available. In total, we found 6 missense variants, 8 frameshift variants, 3 nonsense variants, 1 insertion variant and 2 splice site variants (Table 1 and Fig. 1). Ten of these variants were previously reported (c.43 + 1G > A, p.Leu23Leufs*6, p.Val26Glyfs*28, p.Gly48Valfs*34, c.213-2A > G, p.Tyr73*, p.Glu74_Thr75dup, p.Pro80Gln, p.Arg252* and p.Gln376Pro), and ten novel pathogenic variants were observed (p.Val28Thrfs*3, p.Arg50Trp, p.Pro149Ser, p.Pro151Argfs*6, p.Ala177Thr, p.Pro181Profs*92, p.Glu209Glufs*65, p.Glu302Glufs*11, p.Thr313Ile and p.Tyr325*).

Combined with phenotypes and genotype, the final diagnosis of RCS was established in 9 patients, *PAX2*-related CAKUT was identified in 11 patients and *PAX2*-related nephrosis was identified in 12 patients. None of the missense variants was detected in patients with RCS. Individuals with abnormal kidney structure (RCS or CAKUT group) tended to have a pathogenic LGD variants (*Fisher test*, *p* = 0.02). It did not show significant cluster of variants in the paired domain of *PAX2* among the patients from the different phenotype group (*Fisher test*, *p* > 0.05). Recurrent variants were seen in 16 individuals involved in the paired domain (p.Arg50Trp, p.Val26Glyfs*28, p.Glu74_Thr75dup) or in the transactivation domain (p.Gln376Pro).

### Phenotypic cluster analysis identifies LGD variants of *PAX2* correlated with RCS

**Fig. 2:**
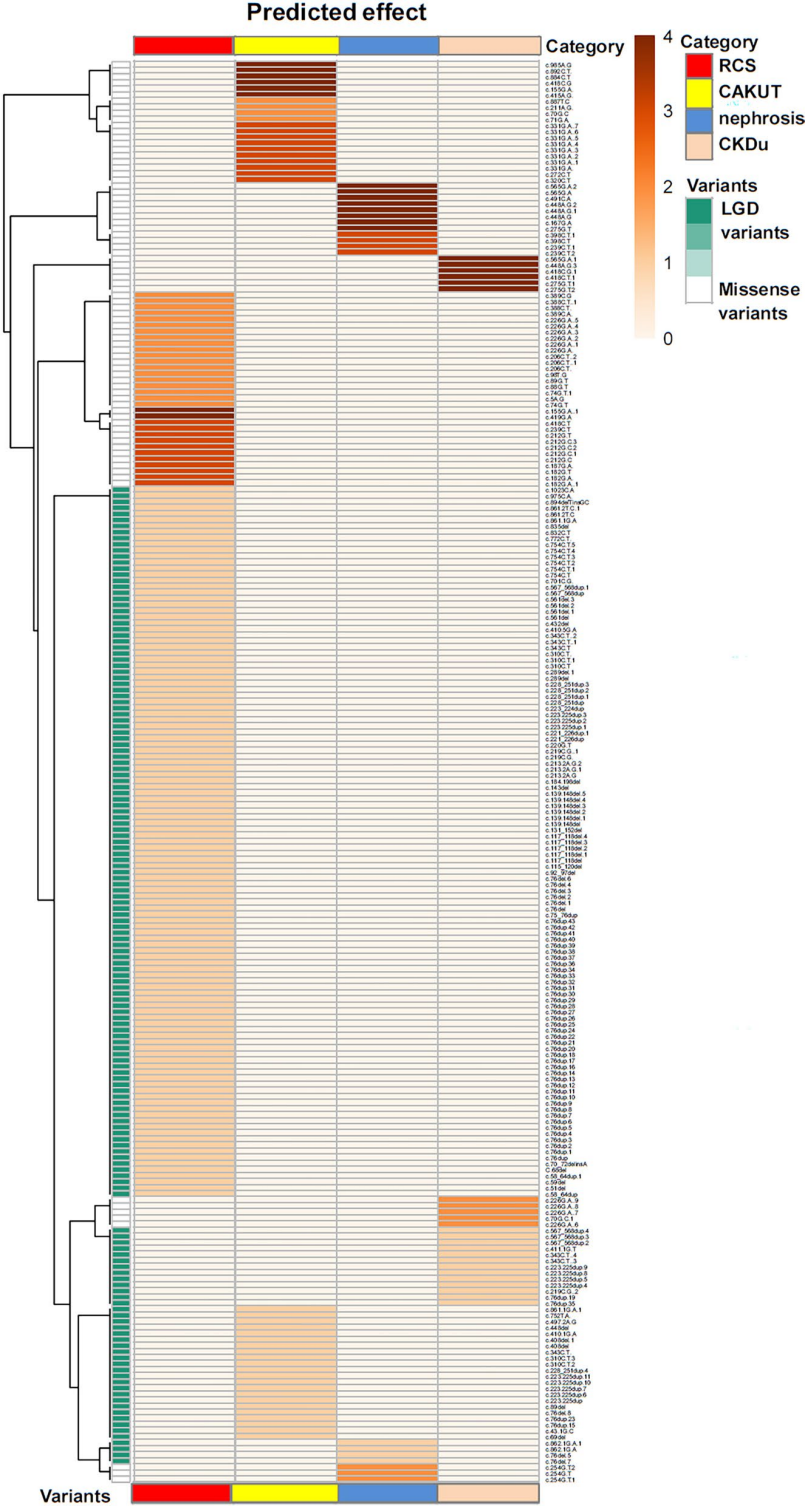
Quantitative phenotype-driven clustering analysis of *PAX2*-associated phenotypes. The clinical features assessed are labeled on the x-axis. Ninety reported pathogenic variants in the available literature from 234 patients with *PAX2*-related kidney disease were enrolled in the clustering analysis. Heat map generated using Manhattan distance with dendrogram and *PAX2* variants shown (right panel)**.** All reported pathogenic missense variants in *PAX2* (white) and a size-matched cohort of likely/presumed gene disruptive (LGD) variants (MediumAquamarine; deletion, truncating, nonsense and frameshift variants with predicted nonsense-mediated decay) were clustered according to phenotypic features using the R packages cluster and gplots, and function heatmap (Red, renal coloboma syndrome, RCS; Yellow, CAKUT, Blue, nephrosis, NavajoWhite, CKD of unknown etiology, CKDu). The clustering reveals that LGD variants are predominantly associated with RCS, whereas missense variants have a wider phenotypic spectrum that includes distinct clusters of CAKUT or nephrosis or CKD of unknown etiology phenotypes. Detailed versions of the phenotyping data set used for clustering analysis and the heat map, with all of the missense and LGD variants labeled, are available (Additional file 1: Table S1)

**Fig. 3:**
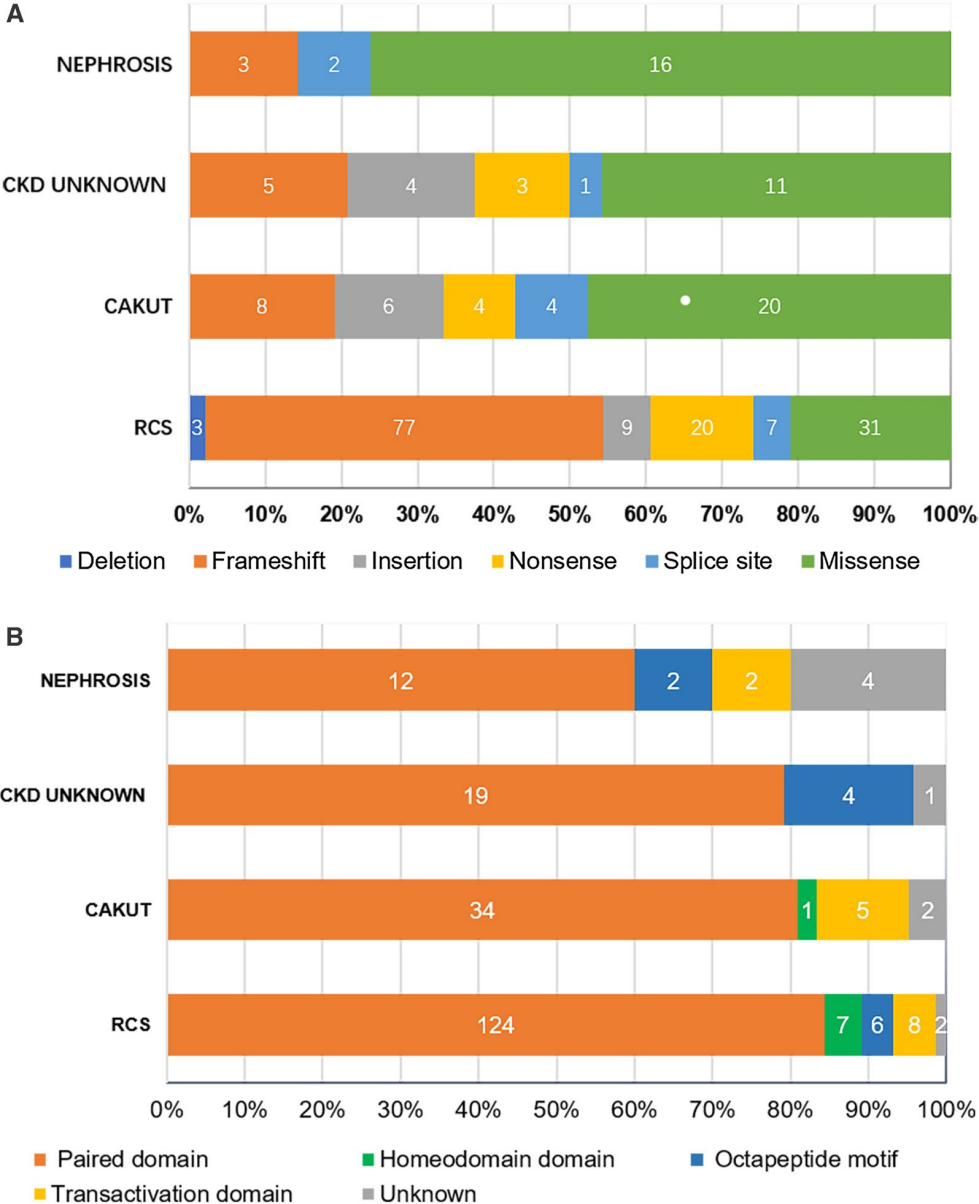
Literature review of all published cases of *PAX2* related disorder. **A** Different types of variants stratified by phenotype in patients with *PAX2*-related disorders. **B** Residuals caused by pathogenic variants within different functional domains stratified by phenotype in patients with *PAX2*-related disorders (Labeled with case number)

To identify pathogenic variants that either mimic haploinsufficiency or represent hypomorphic alleles, we reviewed the phenotypic and genetic data from all international registry sources and published cases that were accessible to us. In the available literature, we found that 90 reported pathogenic variants in *PAX2* from 234 patients with kidney disease (Fig. 1, Additional file 1: Table S1). Among the reported cases of *PAX2*-related disorders, 147/234 (63.0%) individuals were diagnosed with RCS, 42/234 (18.0%) individuals were diagnosed with isolated CAKUT, 19/234 (8.4%) individuals were diagnosed with nephrosis, 2/234 (0.9%) individuals were clinically diagnosed with CAKUT and nephrosis, and 24/234 (10.3%) individuals were screened for genetic cause for CKD of unknown etiology. We performed a cluster analysis to determine whether the missense or LGD variants (variants of deletion, frameshift, insertion, truncating and splice site) in *PAX2* could be distinguished on the basis of their phenotypes. It confirmed that RCS was highly correlated with LGD variants (Fig. 2). There were more cases with missense variants presenting with nephrosis compared the cases with RCS, isolated CAKUT or CKD of unknown (*Fisher test*, *p* < 0.05, Fig. 3). We have noticed 39 patients with non-RCS who was from 156 individuals with LGD variants. Among them, there were 26 individuals who came from the same family with other affected individuals with RCS.

### Protein structural analysis reveals that *PAX2* missense variants affecting DNA binding are highly associated with kidney and ocular development deficiency

To explore the molecular basis underlying the *PAX2* variants, we investigated the protein structural of PAX2 protein including an N-terminal DNA-binding paired domain and a C-terminal transactivation domain (Fig. 1). The location of pathogenic variants was distributed throughout the multiple domains of the PAX2 protein (Fig. 1). There was obvious clustering of variants in the DNA-binding domain from patients with RCS, isolated CAKUT or CKD of unknown etiology compared with those from the patients with nephrosis (*Fisher test*, *p* < 0.05, Fig. 1).

**Fig. 4:**
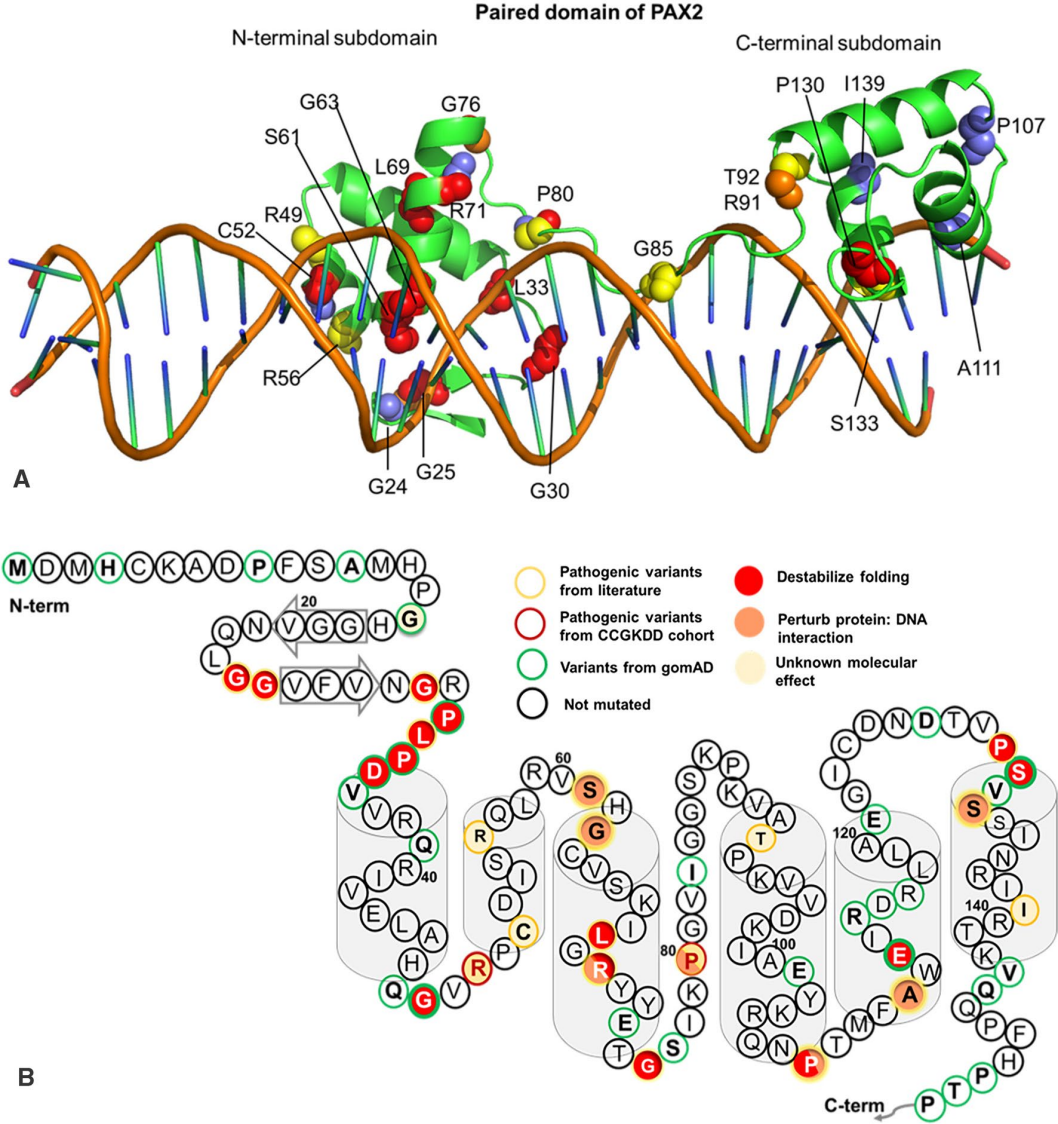
Protein structural analysis and DNA interaction of *PAX2* missense variants. **A** Locations of missense variants stratified by phenotype. The structural analysis of PAX2 was performed using crystallographic structure of PAX5 in complex with DNA (PDB accession 1k78). The sequence of the paired domain of PAX5 differs from that of PAX2 by just three residues (97,122 and 123), all relatively far from those affected by the variants, so that the generation of a homology model was not necessary. Residues caused by pathogenic variants in individuals with renal coloboma syndrome (RCS) were shown in red, residues associated with variants in individuals with isolated CAKUT were shown in yellow, and residues associated with variants in individuals with nephrosis were shown in cornflower blue. Cartoon representation showing both N- and C-terminal domains. The fig was done with the molecular visualization software Pymol. **B** Schematic view of the distribution of *PAX2* variants and the energy results predicted by FoldX. The residues are color coded according to its change in stability. Residues with two colors represent the results for different variants in the same position. Residue numbering throughout the article is based on the Uniprot numbering (Q02962-1, Isoform 1). Protein structure was indicated on base of PAX5 in complex with DNA (PDB accession 1k78) with α-helices shaded in gray cylindrical and β-sheets shaded in gray arrow

Next, the molecular modeling program of FoldX was utilized to predict the effect of *PAX2* missense variants that could disrupt folding or interactions with DNA. We classified all 38 pathogenic missense variants into three categories: those predicted to be highly destabilizing to protein structure and disrupt protein folding (D, n = 15), those predicted to perturb the DNA binding (P, n = 10), and those of unknown effect (U, n = 13). Most of the variants (15/16) identified in RCS group (31 individual) were predicted to affect the DNA binding or the stability of protein folding, compared with only 10/22 from the non-RCS group (47 individuals, *Fisher test*, *p* = 0.002, Fig. 4, Additional file 1: Table S1). Recurrent missense variants were found in three substitutions located in paired domain and were predicted to unstable the protein structure or protein-DNA interaction (p.Arg71Thr, p.Gly76Ser and p.Pro80Leu). Additionally, the missense variants reported from our CCGKDD cohort did not seem to perturb the interaction with DNA (p.Arg50Trp, p.Pro80Gln, p.Pro149Ser, p.Ala177Thr, p.Thr313Ile and p.Gln376Pro). These six missense variants were carried by the patients with nephrosis or isolated CAKUT.

We also investigated the probability of the existing sequence-based phenotype predictors to identify pathogenic *PAX2* missense variants (Table 2). Those predictor tools showed no significant difference in discriminating RCS from non-RCS variants. However, many perform very well at distinguishing the pathogenic *PAX2* missense variants from 223 putatively benign variants present in the gnomAD database (Additional file 1: Table S2). Interestingly, FoldX by use of structure alone, is comparable with the other top-ranking predictors that are largely developed from evolutionary conservation. The results gave evidence of the predicting value on protein structure for the pathogenicity as well as the phenotype of *PAX2* missense variants.

**Table 2:** Ranking computational phenotype predictors for their abilities to distinguish pathogenic *PAX2* missense variants from putatively benign gnomAD variants

Predictor	All pathogenic versus gnomAD			RCS versus non-RCS		
	*P*-value	AUC	95% CI	*P*-value	AUC	95% CI
FoldX (complex)	0	0.752	0.641, 0.864	0.035	0.661	0.463, 0.858
FoldX (monomer)	0.035	0.611	0.496, 0.725	0.0132	0.6645	0.429, 0.792
REVEL	0	0.819	0.734, 0.903	0.02	0.723	0.556, 0.89
Polyphen2	0	0.805	0.689, 0.921	0.152	0.638	0.461, 0.815
VEST4	0	0.779	0.68, 0.879	0.025	0.716	0.548, 0.884
GERP	0	0.735	0.63, 0.84	0.174	0.631	0.454, 0.807
CADD	0	0.716	0.621, 0.811	0.114	0.652	0.475, 0.829
MutationTaster	0.005	0.646	0.556, 0.736	0.315	0.597	0.414, 0.779
MetaSVM	0.26	0.559	0.481, 0.637	0.544	0.442	0.257, 0.627
phyloP30way	0.61	0.527	0.43, 0.624	0.802	0.524	0.339, 0.709
LRT	0	0.281	0.194, 0.368	0.574	0.446	0.261, 0.631
PROVEAN	0	0.222	0.133, 0.312	0.117	0.349	0.169, 0.53
SIFT	0	0.15	0.066, 0.235	0.124	0.352	0.178, 0.527
FATHMM	0	0.146	0.065, 0.227	0.011	0.257	0.095, 0.419

## Discussion

In this study, we explored the phenotypic and genotypic features in a group of 32 children for the presence of heterozygous *PAX2* pathogenic variants. Combining with our research cohort, a system review of 234 reported cases to date with the phenotypic and genotypic information allowed us to define the phenotypic spectrum associated with variants in *PAX2*. The association of typical RCS to heterozygous loss-of-function *PAX2* variants (LGD variants) understandably dominates the human disease literature on *PAX2*-related disorders. Here we put forward an approach to identified a subset of *PAX2* missense variants predicted to affect the protein structure or protein-DNA interaction associated with the phenotype of RCS.

A wide variety of clinical phenotypes have been reported in individuals with *PAX2-*related disorder [8, 9, 11]. Dysplasia of the optic nerve was the main ophthalmological finding of the disorder, which covered 63% of literature-based cases. Among the nine patients with RCS in this study, five patients did not screen for any fundus diseases until identifying the *PAX2* variants, who were subsequently diagnosed with unilateral coloboma. Underdiagnosis of eye lesions in patients might be attributed to the lack of awareness of *PAX2*-related disorder, especially in patients without any complaints of poor eyesight who were diagnosed with unilateral coloboma. The discrepancy in the diagnosis within the same family has been shown in the literature (Additional file 1: Table S1). It may attribute to the phenotypic varieties during the development and the underdiagnosis of the extrarenal anomalies related to *PAX2* variants. Further fundus examination should be conducted for the thirteen patients in current study even with normal visual acuity. It indicated that the genetic diagnosis of *PAX2* pathogenic variants can help to detect the ophthalmological abnormalities early.

The Paired box (Pax) family act as transcription factors that are required for embryonic development through regulating lineage specification and subsequent morphogenesis of tissues and organs [17]. The conservation of the paired domain is identified through phylogenetic analysis (https://www.ncbi.nlm.nih.gov/Structure/cdd/cddsrv.cgi?uid=smart00351) [16], indicating the sequence similarity of the paired domain of *PAX2* with the other PAX family protein. According to the available crystallographic structure of the PAX2 protein, a N-terminal DNA binding paired box domain and C-terminal homeodomain as the second DNA-binding motif is presented in Fig. 1 [9, 15]. In this regard, it is interesting to note that a cluster of variants located in the DNA-binding domain from the patients with development deficiency of kidney or ophthalmology compared that from the patients with nephrosis. We showed that FoldX can be used as a tool to predict DNA-binding specificity on base of the structure of protein–DNA complex. We observed that most of the *PAX2* missense variants in the paired domain leading to kidney and ocular deficiency are driven by the loss of stability of the domain, which is coherent with the early concept that development deficiency was due to haploinsufficiency. Indeed, destabilization of the domain could result in its spatial reorganization and a total loss of binding as a truncated protein would do. The protein structural analysis indicated the correlation between the energetic and structural effects of the missense variants and their phenotypic outcome. Even the previous studies did not reveal a consistent genotype–phenotype correlation [6], we identified a subset of missense variants associated with the phenotype of RCS which perturbs the interaction of the DNA-binding domains. Previous function studies have proved the weak transactivation activity of the *PAX2* dominant variants (p.Arg56Gln, p.Pro80Leu and p.Ser133Phe) was due to decreased proteins-DNA binding [9]. Prediction through the structure-based protein design analysis can help to discriminate between the phenotype of RCS and phenotype of isolated CAKUT or nephrosis. It can allow the accurate prioritization of missense variants in *PAX2* when assessing a potential effect of pathogenic variant detected in utero. Importantly, however, the molecular prediction alone cannot explain all the phenotypic heterogeneity observed among *PAX2* variants. It was highlighted by the fact that there are several examples in our dataset of different patients with the same variant (p.Val26Glyfs*28; p.Thr75dup) exhibiting different phenotypes. Recent studies demonstrated substantial genetic complexity underpinning renal diseases, including some well-documented cases of digenic inheritance [18]. Further work on epigenetic or digenic mechanism could provide novel insights into phenotypic heterogeneity in PAX2 related disorders.

Our study had several limitations. Firstly, we did not report much more extrarenal phenotypes involved in multiple systems (i.e. skeletal deformity, Mullerian duct anomalies, et al.) in our patients. *PAX2* gene is expressed in many tissues besides the kidney and eye, including the optic vesicle, genitourinary tract, pancreas, cerebellum, hypothalamus, and midbrain/hindbrain boundaries [6]. It is important for cell lineage specification in multicellular organisms [19]. A broad variety of non-renal and non-ophthalmological manifestations have been recorded in individuals with *PAX2*-related disorders [3, 11, 20]. We should pay more attention to clinical assessment and tracking of multiple system development in the patient cohort. And deep intronic variants, variants within the variable number tandem repeats and copy number variations in *PAX2* would be explored by genome sequencing to explain the divergent phenotypes. Secondly, we could not predict clinical course and the renal outcome through the genotype lacking of the long-term follow-up information in a large cohort. Further study from the national multicenter registry CCGKDD will be conducted.

## Conclusions

In conclusion, we defined the phenotypic spectrum associated with genotype through analysis on 32 Chinese patients with *PAX2*-related disorder and system review of 234 published cases. The approach shown here predicting the pathogenic variants associated the clinical phenotype could be implemented in a diagnotic strategy for *PAX2*-related disorder. A precise genetic diagnosis at an early stage of the disorder is crucial for the preservation of renal function, optimization of genetic counseling, and improvement of the quality of life of patients.

## Supplementary Information

**Additional file 1**. **Supplementary Table S1.** Genotyping and phenotyping of *PAX2* missense and LGD variants. **Supplementary Table S2.** Protein structural properties and phenotype predictor values for pathogenic and putatively benign PAX2 missense variants.

## Data Availability

The principal datasets generated or analyzed during this study are included in this published article and its supplementary information files. The data generated or analyzed in the current study for the renal coloboma syndrome are available in the NCBI (accession number: MIM #120330, https://www.ncbi.nlm.nih.gov/gene/? term = MIM% 120330). The web links of the relevant datasets were as follows: HGMD, http://www.hgmd.cf.ac.uk/ac), Leiden Open Variation Database (LOVD, http://www.lovd.nl/3.0/http://LOVD.nl/GENE), Clinvar database (https://www.ncbi.nlm.nih.gov/clinvar). Our analysis on the phenotype and genotype is from the database for Chinese children renal disease which is publicly available datasets in the Chinese Children Genetic Kidney Disease Database (CCGKDD, https://www.ccgkdd.com.cn).

## Abbreviations

ACMG: American College of Medical Genetics and Genomics
CAKUT: Congenital anomalies of the kidneys and urinary tract
CCGKDD: Chinese Children Genetic Kidney Disease Database
CKD: Chronic kidney disease
ESRD: End stage renal disease
FSGS: Focal segmental glomerular sclerosis
HGMD: Human Gene Mutation Database
LGD: Likely/presumed gene disruptive
LOVD: Leiden Open Variation Database
PAX2: Paired Box gene 2
RCS: Renal coloboma syndrome
VUR: Vesicoureteral reflux
