# Supplementary material for: Phenotypic spectrum and genetics of PAX2-related disorder in the Chinese cohort
Source: BMC Med Genomics. 2021 Oct 25;14:250. doi: 10.1186/s12920-021-01102-x (PMC8543950; doi:10.1186/s12920-021-01102-x)
Supplement: Supplementary file 1 — Additional file 1. Supplementary Table S1. Genotyping and phenotyping of PAX2 missense and LGD variants. Supplementary Table S2. Protein structural properties and phenotype predictor values for pathogenic and putatively benign PAX2 missense variants. [file 12920_2021_1102_MOESM1_ESM.pdf]

Supplementary Table Table S1. Genotyping and phenotyping of *PAX2* missense and LGD variants

ID	Category	ID2	Ref. PMID	Variants c.change	p change	Variants type	Optical phenotype	Urinalysis	VUR	Kidney development	Renal biopsy	Onset-age	ESRD (age)	other phenotypes
1	CAKUT	CKT-35	Barua et al. (2014) PMID: 24676634	c.5A>G	p.Asp2Gly	missense	No abnormal	NA	Unilateral	NA	NA	2y	NA	NA
2	CAKUT	F1-1	Bower et al. (2012) PMID: 22213154	c.43+1G>C	NA	splice site	No abnormal	No abnormal	NA	bilateral renal hypoplasia	NA	prenatal	no(2y)	No abnormal
3	RCS	SC-184	Rossanti et al. (2020) PMID: 32203253	c.51del	p.His17Glnfs*4	frameshift	Optic disc coloboma	NA	NA	NA	NA	2y	CKD3	No abnormal
4	RCS	10	Okumura et al. (2015) PMID: 26571382	c.58_64dup	p.Gln22Argfs*34	frameshift	bilateral large coloboma involving the entire surface of the optic disc	NA	NA	No abnormal	FSGS	14y	CKD2(14y)	NA
5	RCS	P1	Chung et al. (2001) PMID: 11730657	c.59del	p.Val20Glyfs*9	frameshift	bilateral optic disk coloboma	NA	NA	congenital renal hypoplasia, rickets and	NA	4m	CKD5(HD,5y;Tx	umbilical hernia, right inguinal hernia
6	RCS	10	Okumura et al. (2015) PMID: 26571382	c.58_64dup	p.Gln22Argfs*34	frameshift	bilateral large coloboma involving the entire surface of the optic disc	NA	NA	No abnormal	FSGS	NA	CKD2(14y)	NA
7	CAKUT	P2	Negrisolo et al. (2011) PMID: 21108633	c.69del	p.Val26Cys*3	frameshift	NA	NA	bilateral V	RHD	NA	childhood	CKD5	bilateral sensorineural
8	RCS	985	Cunliffe et al. (1998) PMID: 9783702	C.68del	p.Leu23Profs*6	frameshift	bilateral optic nerve coloboma	No abnormal	left grade	right cystic renal dysplasia	NA	NA	No abnormal	double outlet right ventricle
9	CAKUT	SC-468	Rossanti et al. (2020) PMID: 32203253	c.70G>C	p.Gly24Arg	missense	No abnormal	NA	NA	small cystic kidney	NA	3y	CKD2	Short stature
10	CKD unknov	SC-468 mother	Rossanti et al. (2020) PMID: 32203253	c.70G>C	p.Gly24Arg	missense	NA	NA	NA	No abnormal	NA	32y	CKD	NA
11	RCS	P4	Saida et al. (2020) PMID: 31538321	c.70_72delinsA	p.Gly24Argfs*29	frameshift	bilateral optic-nerve colobomas, visionless left eye	0.5 g/day	grade I VU	No abnormal	FSGS	6y	CKD2-3	NA
12	CAKUT	1	Thomas et al. (2011) PMID: 21380624	c.71G>A	p.Gly24Glu	missense	NA	NA	NA	RHD	NA	15.4y	CKD3	NA
13	RCS	F7	Bower et al. (2012) PMID: 22213154	c.74G>T	p.Gly25Val	missense	bilateral excavation of the papilla and a left colobomatous slit	NA	NA	Medullar and cortical renal cysts. Septated	NA	childhood	CKD2	No abNo abnormal
14	RCS	F7 mother	Bower et al. (2012) PMID: 22213154	c.74G>T	p.Gly25Val	missense	Right optic nerve coloboma	NA	NA	Renal hypoplasia	NA	23y	CKD5(23y)	NA
15	RCS	C-1	Cheong et al. (2007) PMID: 17541647	c.76dup	p.Val26Glyfs*28	frameshift	mild bilateral optic disc colobomas, shallow retinal edema and left macular	proteinuria	No abNo a	bilateral small kidneys, left kidney single cyst	NA	12y	CKD5(HD,13y;	esodeviation
16	RCS	C-2	Cheong et al. (2007) PMID: 17541647	c.76dup	p.Val26Glyfs*28	frameshift	bilateral optic disc colobomas	proteinuria	No abNo a	bilateral small kidneys	NA	8y	CKD5(17y)	nystagmus
17	RCS	C-3	Cheong et al. (2007) PMID: 17541647	c.76dup	p.Val26Glyfs*28	frameshift	optic disc coloboma, left microphthalmia	proteinuria	No abNo a	bilateral small kidneys	FSGS	10y	CKD3(11y)	NA
18	RCS	C-4	Cheong et al. (2007) PMID: 17541647	c.76dup	p.Val26Glyfs*28	frameshift	bilateral mild optic disc colobomas	proteinuria and microscopic hematuria	NA	bilateral renal hypoplasia/atrophy	NA	7y	CKD5(7y; Tx, 9	No abNo abnormal
19	RCS	C-5	Cheong et al. (2007) PMID: 17541647	c.76dup	p.Val26Glyfs*28	frameshift	large optic disc colobomas and chorioretinal degeneration	proteinuria (++)	No abNo a	bilateral, small, echogenic kidneys	NA	1y	no(2.5y)	developmental delay, nystagmus
20	RCS	p5	Fujioka et al. (2011) PMID: 21696512	c.76dup	p.Val26Glyfs*28	frameshift	bilateral megalopapilla with marked excavation and absent central vessels	NA	NA	bilateral renal hypoplasia	NA	neonatal	NA	oligohydramnio,Potter sequence,
21	RCS	P6	Yoshimura et al. (2005) PMID: 15808183	c.76dup	p.Val26Glyfs*28	frameshift	bilateral optic disk coloboma	proteinuria(6y)	NA	bilateral hypoplasia kidneys	NA	3y	CKD3-4(6y)	exotropic
22	RCS	GDA3	Weber et al. (2006) PMID: 16971658	c.76dup	p.Val26Glyfs*28	frameshift	left hypoplastic optic disc,right coloboma	NA	NA	Bilateral dysplasia,single cyst	NA	15y	NA	No abNo abnormal
23	RCS	GDA4	Weber et al. (2006) PMID: 16971658	c.76dup	p.Val26Glyfs*28	frameshift	Optic disc dysplasia	NA	NA	Bilateral hypoplasia	NA	17y	NA	hearing impairment
24	RCS	GDA fathe	Weber et al. (2006) PMID: 16971658	c.76dup	p.Val26Glyfs*28	frameshift	No abNo abnormal	NA	NA	Bilateral hypoplasia	NA	NA	NA	NA
25	RCS	PRA9	Weber et al. (2006) PMID: 16971658	c.76dup	p.Val26Glyfs*28	frameshift	Coloboma, optic disc dysplasia	NA	NA	Bilateral hypoplasia	NA	14y	NA	No abNo abnormal
26	RCS	PRA16	Weber et al. (2006) PMID: 16971658	c.76dup	p.Val26Glyfs*28	frameshift	Coloboma	NA	pyelourete	MCDK	NA	10y	NA	hearing impairment
27	RCS	2	Salomon et al. (2001) PMID: 11168927	c.76dup	p.Val26Glyfs*28	frameshift	papillary dysplasia	NA	No abNo a	renal hypoplasia	oligomeganephr	4y	CKD5(5.5y)	NA
28	RCS	3	Salomon et al. (2001) PMID: 11168927	c.76dup	p.Val26Glyfs*28	frameshift	Coloboma	NA	No abNo a	No abNo abnormal	oligomeganephr	neonatal	CKD5(7y)	NA
29	RCS	SC-607	Rossanti et al. (2020) PMID: 32203253	c.76dup	p.Val26Glyfs*28	frameshift	Optic disc coloboma	NA	NA	cystic kidney	NA	13y	CKD2	Hyperuricemia
30	CAKUT	SC-573	Rossanti et al. (2020) PMID: 32203253	c.76dup	p.Val26Glyfs*28	frameshift	No abNo abnormal	NA	NA	bilateral RHD, RTA	NA	10y	CKD5(Tx,11y)	Autism, median cervical cyst
31	RCS	SC-472	Rossanti et al. (2020) PMID: 32203253	c.76dup	p.Val26Glyfs*28	frameshift	Optic disc coloboma	NA	NA	No abNo abnormal	NA	2y	CKD3	Mild delay in language
32	RCS	SC-472 m	Rossanti et al. (2020) PMID: 32203253	c.76dup	p.Val26Glyfs*28	frameshift	Optic disc coloboma	NA	NA	No abNo abnormal	NA	NA	NA	Urinary lithiasis
33	RCS	SC-415	Rossanti et al. (2020) PMID: 32203253	c.76dup	p.Val26Glyfs*28	frameshift	Optic disc coloboma	NA	NA	No abNo abnormal	NA	26y	CKD5(Tx, 7y)	Small ventricular septal defect
34	CKD unknov	SC-239	Rossanti et al. (2020) PMID: 32203253	c.76dup	p.Val26Glyfs*28	frameshift	No abnormal	NA	NA	No abnormal	NA	2m	CKD	Hypocalcemia,pulmonary artery stenosis
35	RCS	SC-10	Ohtsubo et al. (2012) PMID: 22350371	c.76dup	p.Val26Glyfs*28	frameshift	Optic disc coloboma	mild proteinuria	No abnorr	left renal hypoplasia	NA	6y	CKD3	Scoliosis
36	RCS	SC-10 sis	Ohtsubo et al. (2012) PMID: 22350371	c.76dup	p.Val26Glyfs*28	frameshift	Optic disc coloboma	mild proteinuria	VUR	left renal hypoplasia	glomeruli had dif	4month	no	No abnormal

S			S											
37	RCS	SC-114	Rossanti et al. (2020) PMID: 32203253	c.76dup	p.Val26Glyfs*28	frameshift	Optic disc coloboma	NA	NA	No abnormal	NA	3y	CKD	No abnormal
38	CAKUT	SC-149	Rossanti et al. (2020) PMID: 32203253	c.76dup	p.Val26Glyfs*28	frameshift	No abnormal	NA	bilateral V	No abnormal	NA	9y	CKD3	No abnormal
39	RCS/nephro	1	Iwafuchi et al. (2016) PMID: 27226968	c.76dup	p.Val26Glyfs*28	frameshift	bilateral optic nerve atrophy	proteinuria from 20y 1.6-3.0g/24h,2+,steroid-	No abnorr	slightly small kidneys with bilateral cysts	FSGS(Foot proc	20y	CKD3(52y)	No abnormal
40	RCS	1 son	Iwafuchi et al. (2016) PMID: 27226968	c.76dup	p.Val26Glyfs*28	frameshift	bilateral glaucomatous cupping	proteinuria from 2y	NA	NA	NA	2y	CKD5(PD,5y;T›	NA
41	RCS	1 son2	Iwafuchi et al. (2016) PMID: 27226968	c.76dup	p.Val26Glyfs*28	frameshift	bilateral optic nerve atrophy	proteinuria from birth	NA	bilateral hypoplastic kidneys	NA	neonatal	CKD5(PD,7y; T	No abnormal
42	RCS	1	Sanyanusin et al. (1995b) PMID: 8589702	c.76dup	p.Val26Glyfs*28	frameshift	a bilateral visual field defect with optic nerve colobomas	chronic mild renal failure	No abnorr	NA	NA	NA	NA	NA
43	RCS	2	Sanyanusin et al. (1995b) PMID: 8589702	c.76dup	p.Val26Glyfs*28	frameshift	a bilateral visual field defect with optic nerve colobomas	severe progressive renal failure	No abnorr	NA	NA	NA	CKD5(Tx)	NA
44	RCS	656	Schimmenti et al. (1997) PMID: 9106533	c.76dup	p.Val26Glyfs*28	frameshift	Bilateral opacities of the anterior and posterior lens capsules,hypoplastic	NA	NA	bilateral renal hypoplasia	NA	48y	CKD5(24y)	soft skin
45	RCS	657	Schimmenti et al. (1997) PMID: 9106533	c.76dup	p.Val26Glyfs*28	frameshift	microphthalmos, retrobulbar cyst,optic-nerve aplasia, optic pits, retinochoroidal	NA	NA	bilateral renal hypoplasia	NA	25y	CKD5(Tx, 14y)	soft skin,mentally retarded and
46	RCS	2646	Schimmenti et al. (1997) PMID: 9106533	c.76dup	p.Val26Glyfs*28	frameshift	bilateral optic-nerve colobomas	proteinuria	NA	Renal anomalies	NA	3y	CKD5(Tx, 18y)	high-frequency hearing loss,
47	RCS	f-7	Ford et al. (2001) PMID: 11241473	c.76dup	p.Val26Glyfs*28	frameshift	left macula absence, bilateral abNo abnormal optic discs, optic disc	consistently shown trace or no protein, no	grade III V	bilateral renal hypodysplasia	NA	neonatal	CKD2-3(6y)	nystagmus,left esotropia, nocturnal
48	RCS	f-19	Ford et al. (2001) PMID: 11241473	c.76dup	p.Val26Glyfs*28	frameshift	bilateral optic disc coloboma	NA	bilateral V	No abnormal	NA	4y	CKD5(HD,23y;1	NA
49	RCS	f-21	Ford et al. (2001) PMID: 11241473	c.76dup	p.Val26Glyfs*28	frameshift	bilateral abNo abnormalities of the optic discs	NA	NA	bilateral renal hypoplasia	NA	NA	CKD3（37y）	NA
50	CKD unknov	f-3	Ford et al. (2001) PMID: 11241473	c.76dup	p.Val26Glyfs*28	frameshift	No abnormal	NA	NA	NA	NA	35y	CKD5(HD,60y;1	NA
51	RCS	RA	Amiel et al. (2000) PMID: 11093271	c.76dup	p.Val26Glyfs*28	frameshift	bilateral microphthalmia with right optic nerve dysplasia	NA	NA	Bilateral renal hypoplasia	NA	NA	NA	NA
52	RCS	PE	Amiel et al. (2000) PMID: 11093271	c.76dup	p.Val26Glyfs*28	frameshift	bilateral retinal coloboma	NA	NA	bilateral renal hypoplasia	NA	NA	NA	NA
53	RCS	AR-1	Amiel et al. (2000) PMID: 11093271	c.76dup	p.Val26Glyfs*28	frameshift	bilateral retinal coloboma	NA	NA	Bilateral renal hypoplasia	NA	NA	NA	NA
54	RCS	AR-2	Amiel et al. (2000) PMID: 11093271	c.76dup	p.Val26Glyfs*28	frameshift	bilateral papillary dysplasia	NA	NA	Unilateral renal hypoplasia	NA	NA	NA	NA
55	RCS	AR-3	Amiel et al. (2000) PMID: 11093271	c.76dup	p.Val26Glyfs*28	frameshift	bilateral papillary coloboma	NA	NA	Unilateral renal hypoplasia	NA	NA	NA	NA
56	RCS	8961	Schimmenti et al. (1999) PMID: 10533062	c.76dup	p.Val26Glyfs*28	frameshift	bilateral optic nerve colobomas and other abNo abnormalities limited to the	NA	NA	bilateral renal hypodysplasia	NA	neonatal	CKD5(Tx,3y)	Chiari 1 malformations
57	RCS	F AN_10	Vivante et al. (2019) PMID: 31001663	c.76dup	p.Val26Glyfs*28	frameshift	bilateral coloboma of the optic nerve	nephrotic range proteinuria, urine	NA	mildly echogenic kidneys with No	FSGS	1y	CKD5(4y)	microcephaly, hypertrophy
58	RCS	SC-521	Rossanti et al. (2020) PMID: 32203253	c.76dup	p.Val26Glyfs*28	frameshift	Optic disc coloboma	NA	NA	No abnormal	FSGS	14y	CKD3(14y)	NA
59	RCS	NI	Amiel et al. (2000) PMID: 11093271	c.75_76dup	p.Val26Glyfs*28	frameshift	bilateral optic nerve coloboma	NA	NA	Bilateral renal hypoplasia	NA	NA	NA	NA
60	RCS	SC-287	Rossanti et al. (2020) PMID: 32203253	c.76del	p.Val26Cysfs*3	frameshift	Retinopathy of prematurity	NA	NA	left RHD, left teratoma	NA	1y	CKD5(PD,3y)	Polycystic ovarian disease
61	RCS	SC-252	Rossanti et al. (2020) PMID: 32203253	c.76del	p.Val26Cysfs*3	frameshift	Optic disc coloboma	NA	NA	No abnormal	NA	9y	CKD3	NA
62	RCS	12961	Schimmenti et al. (1999) PMID: 10533062	c.76del	p.Val26Cysfs*3	frameshift	bilateral optic nerve colobomas	NA	VUR	No abnormal	NA	5y	NA	bilateral 4th and 5th digit clinodactyly and
63	RCS	12962	Schimmenti et al. (1999) PMID: 10533062	c.76del	p.Val26Cysfs*3	frameshift	bilateral optic nerve colobomas	NA	VUR(poss	Renal cysts	NA	4y	NA	NA
64	RCS	F1-22	Bekheirnia et al. (2017) PMID: 27657687	c.76del	p.Val26Cysfs*3	frameshift	Optic nerve coloboma and other anomilies	proteinuria	NA	No abnormal	NA	NA	NA	NA
65	nephrosis	F1-24	Bekheirnia et al. (2017) PMID: 27657687	c.76del	p.Val26Cysfs*3	frameshift	NA	proteinuria	NA	No abnormal	NA	NA	NA	NA
66	RCS	F1-32	Bekheirnia et al. (2017) PMID: 27657687	c.76del	p.Val26Cysfs*3	frameshift	Optic nerve coloboma and other anomilies	NA	NA	renal dysplasia	NA	NA	NA	NA
67	nephrosis	F1-33	Bekheirnia et al. (2017) PMID: 27657687	c.76del	p.Val26Cysfs*3	frameshift	NA	proteinuria	NA	No abnormal	membranous ne	NA	NA	NA
68	CAKUT	F1-37	Bekheirnia et al. (2017) PMID: 27657687	c.76del	p.Val26Cysfs*3	frameshift	NA	NA	NA	renal dysplasia	NA	NA	NA	NA
69	RCS	1	Deng et al. (2019) PMID: 31060108	c.88G>T	p.Gly30Cys	missense	No abnormal	Nephrotic-level proteinuria(3.96 g/24hr)	NA	bilateral RHD	NA	9.8y	CKD5(9.8y)	NA
70	RCS	SC-62	Rossanti et al. (2020) PMID: 32203253	c.89G>T	p.Gly30Val	missense	Optic disc coloboma	NA	NA	No abnormal	NA	1y	CKD	NA
71	CAKUT	SC-590	Rossanti et al. (2020) PMID: 32203253	c.89del	p.Gly30Alafs*8	frameshift	No abnormal	NA	NA	bilateral RHD	NA	1y	CKD3	Low set ears
72	RCS	PRA1	Weber et al. (2006) PMID: 16971658	c.92_97del	p.Arg31_Pro32del	deletion	No abnormal	NA	VUR	Bilateral hypoplasia	NA	NA	NA	Hearing impairment
73	RCS	F35	Bower et al. (2012) PMID: 22213154	c.98T>G	p.Leu33Arg	missense	bilateral optic nerve colobomas	NA	VUR	renal hypoplasia, a single renal cyst	NA	NA	CKD5(5y)	NA
74	RCS	1	Salomon et al. (2001) PMID: 11168927	c.115_120del	p.Gln39_Arg 40del	deletion	Coloboma	NA	No abnorr	renal hypoplasia	NA	neonatal	CKD5(20y)	NA

S S

75	RCS	1	Okumura et al. (2015) PMID: 26571382	c.117_118del	p.Arg40Hisfs*13	frameshift	visual disability with some visual field defects,severe left optic nerve	NA	NA	No abnormal	NA	NA	CKD5(HD,44y)	NA
76	RCS	2	Okumura et al. (2015) PMID: 26571382	c.117_118del	p.Arg40Hisfs*13	frameshift	bilateral slight optic nerve coloboma	NA	NA	Malrotation	Glomerulomegal	18y	CKD2(18y)	NA
77	RCS	3	Okumura et al. (2015) PMID: 26571382	c.117_118del	p.Arg40Hisfs*13	frameshift	bilateral slight optic nerve coloboma	NA	NA	Hypoplasia/atrophy	NA	NA	CKD5(HD,61y)	NA
78	RCS	4	Okumura et al. (2015) PMID: 26571382	c.117_118del	p.Arg40Hisfs*13	frameshift	bilateral severe optic nerve coloboma	NA	NA	Malrotation	NA	NA	CKD3(59y)	NA
79	RCS	5	Okumura et al. (2015) PMID: 26571382	c.117_118del	p.Arg40Hisfs*13	frameshift	bilateral slight optic nerve coloboma	NA	NA	Hypoplasia/atrophy	NA	NA	CKD5(HD,61y)	NA
80	RCS	579	Schimmenti et al. (1997) PMID: 9106533	c.131_152del	p.Leu44Profs*32	frameshift	bilateral optic-nerve colobomas	severe proteinuria	NA	Renal hypodysplasia	NA	3m	CKD5(PD,2y)	seizures
81	RCS	II-3	Fletcher et al. (2005) PMID: 16049068	c.139-148del	p.Gln47Glyfs*33	frameshift	optic disc coloboma	mild proteinuria (0.2 g/L)	NA	bilaterally small kidneys with right tubular	medullary spong	21y	CKD4(21y)	bilateral high-tone sensorineural and
82	RCS	III-3	Fletcher et al. (2005) PMID: 16049068	c.139-148del	p.Gln47Glyfs*33	frameshift	bilateral optic disc coloboma	persistent heavy (4+) proteinuria	bilateral g	bilaterally small kidneys	NA	3y	CKD5(HD,14y)	NA
83	RCS	III-4	Fletcher et al. (2005) PMID: 16049068	c.139-148del	p.Gln47Glyfs*33	frameshift	bilateral optic disc coloboma	significant proteinuria	No abnorr	No abnormal	NA	18m	CKD5(24y)	NA
84	RCS	IV-1	Fletcher et al. (2005) PMID: 16049068	c.139-148del	p.Gln47Glyfs*33	frameshift	bilateral optic disc coloboma	No abnormal(3y)	NA	bilateral renal hypoplasia	NA	prenatal	No abnormal(3y	NA
85	RCS	IV-2	Fletcher et al. (2005) PMID: 16049068	c.139-148del	p.Gln47Glyfs*33	frameshift	right optic disc coloboma	No abnormal(8m)	NA	bilaterally small dysplastic kidneys	NA	prenatal	No abnormal(8r	NA
86	RCS	IV-3	Fletcher et al. (2005) PMID: 16049068	c.139-148del	p.Gln47Glyfs*33	frameshift	right optic disc coloboma	significant proteinuria(6m)	NA	small left dysplastic kidney and right MCDK	NA	prenatal	No abnormal(6r	NA
87	RCS	SC-300	Rossanti et al. (2020) PMID: 32203253	c.143del	p.Gly48Vlafs*35	frameshift	bilateral slight optic nerve coloboma	NA	NA	No abnormal	NA	5y	CKD2(5y)	short statue
88	CAKUT	twin1	Iatropoulos et al. (2012) PMID: 22660956	c.155G>A	p.Cys52Tyr	missense	No abnormal	severe proteinuria from 4y	NA	right multicystic kidney, hyperechogenic left	NA	prenatal	CKD5(16y;Tx,2	Joint laxity and skin hyperextensibility
89	RCS	twin2	Iatropoulos et al. (2012) PMID: 22660956	c.155G>A	p.Cys52Tyr	missense	left eye optic nerve coloboma, loss of visual acuity of the left eye	proteinuria 1+(10y), <0.5 g/24 h	NA	hyperechoic renal pyramids at birth. No	NA	neonatal	No abnormal	Joint laxity and skin hyperextensibility
90	nephrosis	FG-IX-1	Barua et al. (2014) PMID: 24676634	c.167G>A	p.Arg56Gln	missense	No abnormal	urine albumin-to-creatinine	NA	NA	NA	36y	NA	NA
91	RCS	F86	Bower et al. (2012) PMID: 22213154	c.182G>A	p.Ser61Asn	missense	bilateral optic nerve coloboma, retinal degeneration of the right eye	NA	NA	bilateral renal hypoplasia	NA	NA	renal insufficien	NA
92	RCS	F86 mothe	Bower et al. (2012) PMID: 22213154	c.182G>A	p.Ser61Asn	missense	bilateral optic nerve coloboma, retinal degeneration of the right eye	NA	NA	NA	NA	NA	CKD5(Tx,50y)	NA
93	RCS	F39	Bower et al. (2012) PMID: 22213154	c.182G>T	p.Ser61Ile	missense	bilateral morning glory syndrome	NA	NA	bilateral renal hypodysplasia	NA	NA	CKD5(9y)	NA
94	RCS	9	Okumura et al. (2015) PMID: 26571382	c.187G>A	p.Gly63Ser	missense	bilateral optic nerve coloboma	NA	NA	Hypoplasia/atrophy	Mes PGN	NA	CKD4(37y)	NA
95	RCS	F40	Bower et al. (2012) PMID: 22213154	c.184-198del	p.His62_Ser66del	deletion	bilateral optic nerve colobomas	NA	VUR	Bilateral renal hypodysplasia with	NA	neonatal	CKD5(1m)	NA
96	RCS	F41	Bower et al. (2012) PMID: 22213154	c.206C>T	p.Leu69Pro	missense	Bilateral optic nerve colobomas.	NA	NA	Bilateral renal hypoplasia	NA	NA	NA	NA
97	RCS	F41 mothe	Bower et al. (2012) PMID: 22213154	c.206C>T	p.Leu69Pro	missense	Bilateral optic nerve colobomas.	NA	NA	Bilateral renal hypoplasia with	NA	NA	NA	short statue
98	RCS	F41 granc	Bower et al. (2012) PMID: 22213154	c.206C>T	p.Leu69Pro	missense	Unilateral optic nerve coloboma.	NA	NA	NA	NA	NA	CKD5	NA
99	CAKUT	A2334-21	Hwang et al. (2014) PMID: 24429398	c.211A>G	p.Arg71Gly	missense	ganglioneuroblastoma	NA	NA	bilateral renal hypodysplasia	NA	NA	NA	NA
100	RCS	O	Higashide et al. (2005) PMID: 15652857	c.212G>C	p.Arg71Thr	missense	bilateral colobomatous disk anomaly and foveal hypoplasia, horizontal	proteinuria(0.1-1.0 g/day)	No abnorr	left renal hypoplasia, right kidney malrolation	cell infiltration in	15y	CKD3(16y)	ventricular septal defect, left thumb
101	RCS	O mother	Higashide et al. (2005) PMID: 15652857	c.212G>C	p.Arg71Thr	missense	coloboma of the choroid and the optic nerve	NA	possibility	No abnormal	NA	childhood	CKD5(39y)	NA
102	RCS	6	Okumura et al. (2015) PMID: 26571382	c.212G>C	p.Arg71Thr	missense	bilateral optic nerve coloboma	NA	NA	Malrotation	TIN	NA	CKD5(HD,34y)	NA
103	RCS	7	Okumura et al. (2015) PMID: 26571382	c.212G>C	p.Arg71Thr	missense	bilateral optic nerve coloboma	NA	NA	Hypoplasia/atrophy	NA	NA	CKD5(HD,61y)	NA
104	RCS	SC-605	Rossanti et al. (2020) PMID: 32203253	c.212G>T	p.Arg71Met	missense	bilateral optic disc coloboma	NA	NA	RHD	NA	2m	CKD4-5	Pulmonary hypertension,
105	RCS	1	Galvez-Ruiz et al. (2017) PMID: 29339962	c.213-2A>G	NA	splice site	bilateral optic nerve coloboma	NA	NA	hypoplastic polycystic kidney disease	NA	childhood	NA	NA
106	RCS	VIL8	Weber et al. (2006) PMID: 16971658	c.213-2A>G	NA	splice site	Optic disc dysplasia	NA	NA	MCDK	NA	NA	NA	NA
107	RCS	VIL8 fathe	Weber et al. (2006) PMID: 16971658	c.213-2A>G	NA	splice site	coloboma	NA	NA	NA	NA	NA	CKD5	NA
108	RCS	F43	Bower et al. (2012) PMID: 22213154	c.219C>G	p.Tyr73*	nonsense	papillary coloboma	NA	NA	bilateral renal hypodysplasia	NA	NA	CKD3-4(17y)	NA
109	RCS	F43 mothe	Bower et al. (2012) PMID: 22213154	c.219C>G	p.Tyr73*	nonsense	unilateral papillary coloboma	NA	NA	bilateral renal hypodysplasia	NA	NA	CKD5(Tx,35y)	NA
110	CKD unknov	F43 granc	Bower et al. (2012) PMID: 22213154	c.219C>G	p.Tyr73*	nonsense	NA	NA	NA	NA	NA	NA	CKD5(Tx,50y)	NA
111	RCS	SC-274	Rossanti et al. (2020) PMID: 32203253	c.220G>T	p.Glu74*	nonsense	Optic disc coloboma	NA	possibility	No abnormal	NA	4y	CKD3(4y)	Retractile testis, mild intellectural
112	RCS	P7	Liu et al. (2018) PMID: 29054766	c.221_226dup	p.Glu74_Thr75dup	insertion	bilateral optic nerve coloboma, right cystic malformation	proteinuria +++, OB +	NA	bilateral renal hypoplasia	NA	10y	CKD5(PD,10y)	nocturnal enuresis from birth, left

S			S											
113	RCS	dup1	Devriendt et al. (1998) PMID: 9760197	c.221_226dup	p.Glu74_Thr75dup	insertion	optic disk dysplasia,papillomacular serous detachment and cystic degeneration of the macula	no proteinuria	VUR	No abnormal	NA	childhood	CKD2-3(17y)	NA
114	CAKUT	F45-1	Bower et al. (2012) PMID: 22213154	c.223-225dup	p.Thr75dup	insertion	No abnormal	NA	NA	Bilateral hypodysplasia, unilateral pyeloureteral	NA	NA	No abnormal(3y	NA
115	RCS	F45-2	Bower et al. (2012) PMID: 22213154	c.223-225dup	p.Thr75dup	insertion	small papilla with edges not well defined, three microcoloboma at the temporal part of one retina	NA	NA	bilateral hypodysplasia	NA	NA	No abnormal(3(	NA
116	RCS	F45-3	Bower et al. (2012) PMID: 22213154	c.223-225dup	p.Thr75dup	insertion	small papilla with edges not well defined, one microcoloboma at the peripheral part of one retina	NA	NA	hypodysplasia	NA	NA	CKD	NA
117	RCS	F45-4	Bower et al. (2012) PMID: 22213154	c.223-225dup	p.Thr75dup	insertion	small coloboma at the temporal side of one retina	NA	NA	NA	NA	NA	CKD5(36y)	NA
118	CKD unknov	F45-5	Bower et al. (2012) PMID: 22213154	c.223-225dup	p.Thr75dup	insertion	NA	NA	NA	NA	NA	NA	CKD5(12y)	NA
119	CKD unknov	F45-6	Bower et al. (2012) PMID: 22213154	c.223-225dup	p.Thr75dup	insertion	NA	NA	NA	NA	NA	NA	CKD5(Tx,19y)	NA
120	CAKUT	F45-7	Bower et al. (2012) PMID: 22213154	c.223-225dup	p.Thr75dup	insertion	NA	NA	VUR	hypodysplasia	NA	NA	NA	NA
121	CAKUT	F45-8	Bower et al. (2012) PMID: 22213154	c.223-225dup	p.Thr75dup	insertion	NA	NA	VUR	single kidney with hypodysplasia	NA	NA	NA	NA
122	CKD unknov	F45-9	Bower et al. (2012) PMID: 22213154	c.223-225dup	p.Thr75dup	insertion	NA	NA	NA	NA	NA	NA	CKD5(60y)	NA
123	CKD unknov	F45-10	Bower et al. (2012) PMID: 22213154	c.223-225dup	p.Thr75dup	insertion	NA	NA	NA	NA	NA	NA	CKD5(79y)	NA
124	CAKUT	F45-11	Bower et al. (2012) PMID: 22213154	c.223-225dup	p.Thr75dup	insertion	NA	NA	NA	bilateral hypodysplasia	NA	NA	CKD	NA
125	CAKUT	F45-12	Bower et al. (2012) PMID: 22213154	c.223-225dup	p.Thr75dup	insertion	NA	NA	VUR	single kidney with hypodysplasia	NA	NA	CKD5(19y;Tx,2(	NA
126	RCS	11	Okumura et al. (2015) PMID: 26571382	c.223_224dup	p.Gly76Profs*8	frameshift	bilateral optic nerve coloboma	NA	NA	Hypoplasia/atrophy	FSGS	NA	CKD5(HD,38y)	NA
127	RCS	V:2	Devriendt et al. (1998) PMID: 9760197	c.226G>A	p.Gly76Ser	missense	bilateral optic disk dysplasia with optic pits and unusual pattern of retinal	NA	NA	renal hypoplasia	NA	16y	CKD2(16y)	NA
128	RCS	IV:2	Devriendt et al. (1998) PMID: 9760197	c.226G>A	p.Gly76Ser	missense	bilateral peripapillary atrophy and unusual pattern of retinal vessels	NA	NA	No abnormal	NA	NA	CKD5(32y)	NA
129	RCS	IV:3	Devriendt et al. (1998) PMID: 9760197	c.226G>A	p.Gly76Ser	missense	mild form of optic disk anomaly with unusual pattern of retinal vessels	asymptomatic proteinuria	NA	Unilateral renal extremely hypoplasia	NA	NA	CKD4(46y)	NA
130	RCS	IV:6	Devriendt et al. (1998) PMID: 9760197	c.226G>A	p.Gly76Ser	missense	right optic pits and large exacvation, bilateral unusual pattern of retinal vessels	asymptomatic proteinuria	NA	renal hypoplasia	NA	NA	CKD5(33y)	NA
131	RCS	IV:7	Devriendt et al. (1998) PMID: 9760197	c.226G>A	p.Gly76Ser	missense	right optic pits and bilateral unusual pattern of retinal vessels	asymptomatic proteinuria	NA	No abnormal	NA	NA	CKD5(22y)	NA
132	RCS	III:8	Devriendt et al. (1998) PMID: 9760197	c.226G>A	p.Gly76Ser	missense	bilateral optic nerve coloboma	NA	NA	bilateral renal hypoplasia	NA	NA	CKD3(70y)	NA
133	CKD unknov	III:6	Devriendt et al. (1998) PMID: 9760197	c.226G>A	p.Gly76Ser	missense	NA	NA	NA	NA	NA	NA	CKD5(censorec	NA
134	CKD unknov	III:4	Devriendt et al. (1998) PMID: 9760197	c.226G>A	p.Gly76Ser	missense	NA	NA	NA	NA	NA	NA	CKD5(censorec	NA
135	CKD unknov	II:3	Devriendt et al. (1998) PMID: 9760197	c.226G>A	p.Gly76Ser	missense	NA	NA	NA	NA	NA	NA	CKD5(censorec	NA
136	CKD unknov	II:4	Devriendt et al. (1998) PMID: 9760197	c.226G>A	p.Gly76Ser	missense	NA	NA	NA	NA	NA	NA	CKD5(censorec	NA
137	RCS	I:1	Adam et al. (2013) PMID: 27293569	c.228_251dup	p.Ser77_Gly84dup	insertion	bilateral optic coloboma	proteinuria	NA	bilateral RHD	NA	NA	CKD4（62y）	high-frequency hearing loss, gout
138	RCS	II:1	Adam et al. (2013) PMID: 27293569	c.228_251dup	p.Ser77_Gly84dup	insertion	unilaternal optic nerve hypoplasia, papilloedema	severe proteinuria (24 h urinary protein 4.4 g)	NA	bilateral RHD	tubular atrophy, i	NA	CKD5(HD,39y)	No abnormal
139	RCS	II:2	Adam et al. (2013) PMID: 27293569	c.228_251dup	p.Ser77_Gly84dup	insertion	bilateral optic disc pits, central serous chorioretinopathy	No abnormal	No abnorr	No abnormal	NA	NA	No abnormal	No abnormal
140	RCS	II:3	Adam et al. (2013) PMID: 27293569	c.228_251dup	p.Ser77_Gly84dup	insertion	cupped optic disc and mildly tortuous blood vessels	mild proteinuria	NA	No abnormal	NA	NA	No abnormal	No abnormal
141	CAKUT	III:1	Adam et al. (2013) PMID: 27293569	c.228_251dup	p.Ser77_Gly84dup	insertion	NA	proteinuria	VUR	No abnormal	mesangiocapilla	2y	CKD5(17y;Tx,1	hypospadias
142	RCS	SC-183	Rossanti et al. (2020) PMID: 32203253	c.239C>T	p.Pro80Leu	missense	Optic disc coloboma	NA	NA	No abnormal	NA	14y	CKD2（14y）	NA
143	nephrosis	FG-DG-1	Barua et al. (2014) PMID: 24676634	c.239C>T	p.Pro80Leu	missense	No abnormal	proteinuria	NA	NA	NA	7y	NA	NA
144	nephrosis	FG-DG-2	Barua et al. (2014) PMID: 24676634	c.239C>T	p.Pro80Leu	missense	No abnormal	proteinuria	NA	NA	NA	11y	NA	NA
145	nephrosis	F A4041 p	Vivante et al. (2019) PMID: 31001663	c.254G>T	p.Gly85Val	missense	No abnormal	nephrotic range proteinuria	NA	No abnormal	FSGS	NA	CKD3(13y)	NA
146	nephrosis	F A4041 s	Vivante et al. (2019) PMID: 31001663	c.254G>T	p.Gly85Val	missense	No abnormal	nephrotic range proteinuria	NA	No abnormal	FSGS	NA	CKD2(10y)	NA
147	nephrosis	F A4041 f	Vivante et al. (2019) PMID: 31001663	c.254G>T	p.Gly85Val	missense	No abnormal	nephrotic range proteinuria	NA	No abnormal	NA	NA	CKD5(39y;Tx,4(	NA

S **S**

148	nephrosis/C/	6	Deng et al. (2019) PMID: 31060108	c.272C>T	p.Ala91Val	missense	NA	proteinuria(0.43 g/24hr)	NA	Bilateral renal hypoplasia, left single	focal proliferative	9.7y	CKD3(11y)	fourth metatarsal microsomia of
149	nephrosis	F A5281 p	Vivante et al. (2019) PMID: 31001663	c.275G>T	p.Thr92Met	missense	NA	nephrotic range proteinuria	NA	No abnormal	FSGS	18y	No abnormal	Cryptorchidism
150	CKD unknov	F A5281 n	Vivante et al. (2019) PMID: 31001663	c.275G>T	p.Thr92Met	missense	NA	NA	NA	NA	NA	NA	CKD5(50y)	NA
151	CKD unknov	F A5281 g	Vivante et al. (2019) PMID: 31001663	c.275G>T	p.Thr92Met	missense	NA	NA	NA	NA	NA	NA	CKD5(70y)	NA
152	RCS	MA-1	Amiel et al. (2000) PMID: 11093271	c.289del	p.Asp97Thrfs*62	frameshift	bilateral optic nerve coloboma and severe myopia	NA	NA	Unilateral renal agenesis	NA	NA	NA	NA
153	RCS	MA-2	Amiel et al. (2000) PMID: 11093271	c.289del	p.Asp97Thrfs*62	frameshift	bilateral optic nerve coloboma	NA	NA	slight reduction in kidney size	NA	NA	CKD	NA
154	RCS	SC-315	Rossanti et al. (2020) PMID: 32203253	c.310C>T	p.Arg104*	nonsense	Optic disc coloboma	NA	NA	No abnormal	NA	6m	CKD5(Tx,2y)	left congenital cystic adenomatoid
155	RCS	SC-47	Rossanti et al. (2020) PMID: 32203253	c.310C>T	p.Arg104*	nonsense	Optic disc coloboma	NA	NA	bilateral RHD	NA	2m	CKD4(2m)	NA
156	CAKUT	SC-47 mc	Rossanti et al. (2020) PMID: 32203253	c.310C>T	p.Arg104*	nonsense	No abnormal	NA	NA	bilateral RHD	NA	NA	NA	NA
157	CAKUT	SC-47 brc	Rossanti et al. (2020) PMID: 32203253	c.310C>T	p.Arg104*	nonsense	No abnormal	NA	NA	bilateral RHD	NA	2m	NA	NA
158	RCS	C-6	Cheong et al. (2007) PMID: 17541647	c.310C>T	p.Arg104*	nonsense	mild bilateral optic disc colobomas	proteinuria, microscopic hematuria	No abnorr	bilateral renal hypodysplasia,	NA	4m	CKD5（Tx,10y）	nystagmus，hearing loss,developmental
159	CAKUT	A1087-21	Hwang et al. (2014) PMID: 24429398	c.320C>T	p.Pro107Leu	missense	NA	NA	bilateral u	No abnormal	NA	NA	NA	NA
160	CAKUT	R-32	Sellick et al. (2004) PMID: 15561999	c.331G>A	p.Ala111Thr	missense	No abnormal	NA	NA	bilateral renal agenesis to renal dysplasia	NA	NA	NA	NA
161	CAKUT	R-35	Sellick et al. (2004) PMID: 15561999	c.331G>A	p.Ala111Thr	missense	No abnormal	NA	NA	bilateral renal agenesis to renal dysplasia	NA	NA	NA	NA
162	CAKUT	R-36	Sellick et al. (2004) PMID: 15561999	c.331G>A	p.Ala111Thr	missense	No abnormal	NA	NA	bilateral renal agenesis to renal dysplasia	NA	NA	NA	NA
163	CAKUT	R-38	Sellick et al. (2004) PMID: 15561999	c.331G>A	p.Ala111Thr	missense	No abnormal	NA	NA	bilateral renal agenesis to renal dysplasia	NA	NA	NA	NA
164	CAKUT	R-41	Sellick et al. (2004) PMID: 15561999	c.331G>A	p.Ala111Thr	missense	No abnormal	NA	NA	bilateral renal agenesis to renal dysplasia	NA	NA	NA	NA
165	CAKUT	R-42	Sellick et al. (2004) PMID: 15561999	c.331G>A	p.Ala111Thr	missense	No abnormal	NA	NA	bilateral renal agenesis to renal dysplasia	NA	NA	NA	NA
166	CAKUT	R-46	Sellick et al. (2004) PMID: 15561999	c.331G>A	p.Ala111Thr	missense	No abnormal	NA	NA	bilateral renal agenesis to renal dysplasia	NA	NA	NA	NA
167	CAKUT	R-47	Sellick et al. (2004) PMID: 15561999	c.331G>A	p.Ala111Thr	missense	No abnormal	NA	NA	bilateral renal agenesis to renal dysplasia	NA	NA	NA	NA
168	RCS	SC-622	Rossanti et al. (2020) PMID: 32203253	c.343C>T	p.Arg104*	nonsense	optic disc coloboma, right orbital cyst	NA	NA	No abnormal	NA	1m	CKD4	Micrognathia
169	CAKUT	A3872-21	Hwang et al. (2014) PMID: 24429398	c.343C>T	p.Arg104*	nonsense	NA	NA	NA	bilateral renal hypodysplasia	NA	NA	NA	NA
170	RCS	UM073-35	Schimmenti et al. (2003) PMID: 14566649	c.343C>T	p.Arg104*	nonsense	optic nerve dysgenesis, a large and deep optic nerve cup, tortuous retinal	NA	NA	No abnormal	chronic tubular ir	20y	CKD5(Tx,25y)	high-frequency hearing loss
171	RCS	UM073-22	Schimmenti et al. (2003) PMID: 14566649	c.343C>T	p.Arg104*	nonsense	retinal pigment epithelium thinning,atrophy of the optic nerve	NA	NA	NA	NA	19y	CKD5(Tx,60y)	high-frequency hearing loss
172	CKD unknov	UM073-29	Schimmenti et al. (2003) PMID: 14566649	c.343C>T	p.Arg115*	nonsense	NA	NA	NA	NA	NA	NA	CKD5(censorec	NA
173	CKD unknov	UM073-21	Schimmenti et al. (2003) PMID: 14566649	c.343C>T	p.Arg115*	nonsense	NA	NA	NA	NA	NA	NA	CKD5(censorec	NA
174	RCS	M-1	Miyazawa et al. (2009) PMID: 19954729	c.389C>A	p.Pro130His	missense	optic nerve coloboma	Proteinuria 1.0 to 1.5 g/day	NA	NA	NA	NA	CKD5(12y)	mild developmental delay
175	RCS	F56	Bower et al. (2012) PMID: 22213154	c.388C>T	p.Pro130Ser	missense	AbNo abnormal left optic disc	NA	VUR	No abnormal	FSGS	20y	CKD5(20y)	NA
176	RCS	F56 granc	Bower et al. (2012) PMID: 22213154	c.388C>T	p.Pro130Ser	missense	left optic disc coloboma	NA	NA	Bilateral renal dysplasia	NA	2y	NA	Horizontal nystagmus
177	RCS	2	Galvez-Ruiz et al. (2017) PMID: 29339962	c.389C>G	p.Pro130Arg	missense	bilateral optic nerve abNo abnormalities with central excavations	NA	NA	polycystic kidney disease	NA	NA	CKD5(Tx,24y)	NA
178	nephrosis	FG-GE-1	Barua et al. (2014) PMID: 24676634	c.398C>T	p.Ser133Phe	missense	No abnormal	proteinuria	NA	Slightly small kidney, calyceal diverticulum	FSGS	NA	CKD5	NA
179	nephrosis	FG-GE-2	Barua et al. (2014) PMID: 24676634	c.398C>T	p.Ser133Phe	missense	No abnormal	proteinuria	NA	NA	NA	NA	NA	NA
180	CAKUT	A1743-12	Hwang et al. (2014) PMID: 24429398	c.408del	p.Asn136Lysfs*23	frameshift	NA	NA	NA	renal cysts	NA	NA	NA	NA
181	CAKUT	A1743-21	Hwang et al. (2014) PMID: 24429398	c.408del	p.Asn136Lysfs*23	frameshift	NA	NA	NA	renal cysts	NA	NA	NA	NA
182	CAKUT	10	Deng et al. (2019) PMID: 31060108	c.410+1G>A	NA	splice site	No abnormal	Nephrotic-level proteinuria, microscopic	NA	Unilateral renal hypoplasia, right single	NA	neonatal	CKD2(neonatal	NA
183	RCS	1	Negrisolo et al. (2011) PMID: 21108633	c.410+5G>A	NA	splice site	bilateral excavation of the optic disk	NA	NA	renal hypoplasia, oligomeganephronia	oligomeganephr	NA	NA	NA
184	CKD unknov	3	Thomas et al. (2011) PMID: 21380624	c.411-1G>T	NA	splice site	NA	NA	NA	NA	NA	NA	CKD3(13.5y)	NA
185	CAKUT	CKT-34C	Barua et al. (2014) PMID: 24676634	c.415A>G	p.Ile139Val	missense	No abnormal	NA	Unilateral	No abnormal	NA	NA	NA	dysmorphic facial syndrome,

S			S											
186	RCS	1	Rachwani et al. (2019) PMID: 31692565	c.418C>T	p.Arg140Trp	missense	optic disc coloboma, retinal vasculature with multiple cilioretinal	proteinuria (750mg/day)	NA	RHD, oligomeganephronia	FSGS secondary	14y	No abnormal	cryptorchidism, phimosis and
187	CKD unknov	SC-351	Rossanti et al. (2020) PMID: 32203253	c.418C>T	p.Arg140Trp	missense	No abnormal	NA	NA	No abnormal	NA	NA	CKD3	Hyponatremia
188	CAKUT	pro	Zhang et al. (2018) PMID: 30241513	c.418C>G	p.Arg140Gly	missense	No abnormal	protienuria	NA	Bilateral renal hypoplasia	NA	prenatal	CKD3(3y)	NA
189	CKD unknov	father	Zhang et al. (2018) PMID: 30241513	c.418C>G	p.Arg140Gly	missense	No abnormal	NA	NA	NA	NA	NA	CKD5(Tx,20y)	NA
190	RCS	SC-553	Rossanti et al. (2020) PMID: 32203253	c.419G>A	p.Arg140Gln	missense	Optic disc coloboma	NA	NA	bilateral RHD	NA	12y	CDK4-5(12y)	NA
191	RCS	SC-32	Rossanti et al. (2020) PMID: 32203253	c.432del	p.Gln144Hisfs*15	frameshift	Optic disc coloboma	NA	NA	bilateral RHD	NA	5m	CKD4(5m)	NA
192	nephrosis	FG-JO-1	Barua et al. (2014) PMID: 24676634	c.448A>G	p.Thr150Ala	missense	No abnormal	NA	NA	NA	FSGS	31.5y	CKD5(30-36y)	NA
193	nephrosis	FG-JO-2	Barua et al. (2014) PMID: 24676634	c.448A>G	p.Thr150Ala	missense	NA	NA	NA	NA	FSGS	NA	CKD5(30-37y)	NA
194	nephrosis	FG-JO-3	Barua et al. (2014) PMID: 24676634	c.448A>G	p.Thr150Ala	missense	No abnormal	NA	NA	NA	FSGS	NA	CKD5(30-38y)	NA
195	CKD unknov	FG-JO-4	Barua et al. (2014) PMID: 24676634	c.448A>G	p.Thr150Ala	missense	NA	NA	NA	NA	NA	NA	CKD5(30-38y)	NA
196	CAKUT	F62	Bower et al. (2012) PMID: 22213154	c.448del	p.Thr150Argfs*9	frameshift	No abnormal	NA	NA	Bilateral renal hypoplasia	Oligomeganephr	6y	CKD5(6y)	NA
197	nephrosis	FG-BF-1	Barua et al. (2014) PMID: 24676634	c.491C>A	p.Thr164Asn	missense	No abnormal	NA	NA	Increased echogenicity	FSGS	8y	CKD5	NA
198	CAKUT	SC-30	Rossanti et al. (2020) PMID: 32203253	c.497-2A>G	NA	splice site	No abnormal	NA	NA	right MCDK, left RHD	NA	2y	CKD5(Tx,7y)	Autism,congenital cystic adenomatoid
199	RCS	1	Sanyanusin et al. (1996) PMID: 7795640	c.561del	p.Asn188Metfs*40	frameshift	bilateral optic nerve coloboma	chronic mild proteinuria without hematuria	bilateral g	non-function right kidney, bilateral renal	NA	18m	CKD5(15y)	nystagmus, strabismus, short
200	RCS	2	Sanyanusin et al. (1996) PMID: 7795640	c.561del	p.Asn188Metfs*40	frameshift	bilateral optic nerve coloboma	NA	grade II V	renal hypoplasia, poor corticomedullary	NA	NA	mild renal dysfu	NA
201	RCS	3	Sanyanusin et al. (1996) PMID: 7795640	c.561del	p.Asn188Metfs*40	frameshift	bilateral optic nerve coloboma	chronic mild proteinuria without hematuria	grade I V	bilateral hypoplastic kidneys, diffuse	NA	10w	CKD5(Tx,5y)	short stature
202	RCS	4	Sanyanusin et al. (1996) PMID: 7795640	c.561del	p.Asn188Metfs*40	frameshift	bilateral optic nerve coloboma	chronic mild proteinuria without hematuria	NA	No abnormal	NA	childhood	CKD3(35y)	scleral staphyloma
203	nephrosis	FG-EQ-1	Barua et al. (2014) PMID: 24676634	c.565G>A	p.Gly189Arg	missense	No abnormal	NA	NA	bilateral renal pelvis dilatation	FSGS	17y	CKD5(40y)	NA
204	CKD unknov	FG-EQ-2	Barua et al. (2014) PMID: 24676634	c.565G>A	p.Gly189Arg	missense	No abnormal	NA	NA	No abnormal	NA	NA	CKD5(58y)	NA
205	nephrosis	FG-EQ-4	Barua et al. (2014) PMID: 24676634	c.565G>A	p.Gly189Arg	missense	No abnormal	indeterminate	NA	No abnormal	NA	NA	No abnormal	NA
206	RCS	1	Megaw et al. (2013) PMID: 23686327	c.567_568dup	p.Ile190Argfs*39	frameshift	bilateral optic coloboma	NA	NA	renal hypoplasia	NA	12y	NA	nystagmus, bilateral cryptorchidism, gout,
207	RCS	1 father	Megaw et al. (2013) PMID: 23686327	c.567_568dup	p.Ile190Argfs*39	frameshift	bilateral coloboma	NA	NA	renal hypoplasia	NA	NA	NA	insulin-controlled diabetes, gout and
208	CKD unknov	1 uncle	Megaw et al. (2013) PMID: 23686327	c.567_568dup	p.Ile190Argfs*39	frameshift	blind	NA	NA	NA	NA	NA	CKD5(censorec	gout
209	CKD unknov	1 uncle2	Megaw et al. (2013) PMID: 23686327	c.567_568dup	p.Ile190Argfs*39	frameshift	blind	NA	NA	NA	NA	NA	CKD5(censorec	gout
210	CKD unknov	1 grandp	Megaw et al. (2013) PMID: 23686327	c.567_568dup	p.Ile190Argfs*39	frameshift	blind	NA	NA	NA	NA	NA	CKD5(censorec	gout
211	RCS	F66-1	Bower et al. (2012) PMID: 22213154	c.701C>G	p.234Ser*	nonsense	bilateral optic nerve coloboma	NA	NA	Bilateral renal hypoplasia, left	NA	NA	NA	Cerebral ventriculomegaly
212	CAKUT	P	Nicolaou et al. (2016) PMID: 26489027	c.752T>A	p.251Leu*	nonsense	No abnormal	NA	NA	unilateral renal dysplasia	NA	NA	NA	NA
213	RCS	I-1	Porteous et al. (2000) PMID: 10587573	c.754C>T	p.252Arg*	nonsense	optic pit right eye, optic nerve coloboma left eye	NA	No abnorr	No abnormal	NA	NA	CKD3(70y)	NA
214	RCS	II-5	Porteous et al. (2000) PMID: 10587573	c.754C>T	p.252Arg*	nonsense	bilateral optic nerve coloboma	NA	NA	bilateral small kidneys, cortical	NA	NA	CKD5(35y)	NA
215	RCS	II-9	Porteous et al. (2000) PMID: 10587573	c.754C>T	p.252Arg*	nonsense	bilateral optic nerve coloboma	NA	NA	bilateral nephrolithiasis, single functioning right	NA	NA	CKD3(39y)	NA
216	RCS	III-13	Porteous et al. (2000) PMID: 10587573	c.754C>T	p.252Arg*	nonsense	bilateral optic nerve coloboma and hypoplastic optic nerve in left eye	NA	NA	bilateral small kidneys with cystic apperance	NA	NA	CKD5(21y)	NA
217	RCS	III-16	Porteous et al. (2000) PMID: 10587573	c.754C>T	p.252Arg*	nonsense	bilateral optic nerve pits	NA	VUR	No abnormal	NA	NA	No abnormal	NA
218	RCS	III-17	Porteous et al. (2000) PMID: 10587573	c.754C>T	p.252Arg*	nonsense	bilateral optic nerve coloboma	NA	NA	bilateral small kidneys, cortical	NA	NA	CKD5(6y)	NA
219	RCS	F68-1	Bower et al. (2012) PMID: 22213154	c.772C>T	p.258Gln*	nonsense	Optic nerve coloboma	NA	NA	No abnormal	NA	NA	CKD5	NA
220	RCS	SC-456	Rossanti et al. (2020) PMID: 32203253	c.832C>T	p.258Gln*	nonsense	Optic disc coloboma	NA	NA	bilateral RHD	NA	14y	CKD4-5(14y)	NA
221	RCS	F70	Bower et al. (2012) PMID: 22213154	c.835del	p.Ala279Hisfs*18	frameshift	bilateral optic nerve coloboma	NA	NA	Hypoplastic kidney; dysplastic kidney	NA	NA	CKD5(Tx,13y)	NA
222	RCS	F71-1	Bower et al. (2012) PMID: 22213154	c.861+1G>A	NA	splice site	unilateral optic nerve coloboma	NA	NA	severe renal hypoplasia without evidence of	NA	GA	prenatal Oligoa	NA
223	CAKUT	F71-2	Bower et al. (2012) PMID: 22213154	c.861+1G>A	NA	splice site	NA	NA	VUR	renal hypoplasia, VUR	NA	NA	CKD5(Tx,26y)	unicornate uterus

S			S											
224	RCS	2 mother	Martinovic-Bouriel et al. (2010)	c.861+2T>C	NA	splice site	retinal coloboma and macular detachment	NA	NA	NA	NA	NA	CKD5(HD,17y;T	NA
225	RCS	2 grandf	Martinovic-Bouriel et al. (2010)	c.861+2T>C	NA	splice site	retinal coloboma and macular detachment	NA	NA	NA	NA	NA	CKD5(Tx,29y)	NA
226	nephrosis	F A5089	Vivante et al. (2019) PMID: 31001663	c.862-1G>A	NA	splice site	No abnormal	nephrotic range proteinuria	NA	No abnormal	FSGS	NA	CKD5(27y)	NA
227	nephrosis	F A5089 f	Vivante et al. (2019) PMID: 31001663	c.862-1G>A	NA	splice site	No abnormal	nephrotic range proteinuria	NA	No abnormal	NA	NA	No abnormal	NA
228	CAKUT	CKT-89C	Barua et al. (2014) PMID: 24676634	c.884C>T	p.Ala295Val	missense	NA	NA	Left UPJC	No abnormal	NA	NA	NA	NA
229	CAKUT	CKT-46C	Barua et al. (2014) PMID: 24676634	c.887T>C	p.Leu296Pro	missense	NA	NA	NA	horseshoe, small ectopic left kidney	NA	NA	NA	Multiple congenital abNo abnormalities
230	CAKUT	CKT-39C	Barua et al. (2014) PMID: 24676634	c.892C>T	p.Pro298Ser	missense	NA	NA	NA	Solitary kidney and mild unilateral	NA	NA	NA	NA
231	RCS	F73	Bower et al. (2012) PMID: 22213154	c.894delTinsGC	p.Gly299Argfs*3	frameshift	bilateral optic nerve coloboma	NA	grade II V	Bilateral echogenic kidneys	NA	prenatal	CKD	strabismus; nasolacrimal duct
232	RCS	F75-1	Bower et al. (2012) PMID: 22213154	c.975C>A	p.326Tyr*	nonsense	bilateral optic nerve coloboma	NA	NA	Renal hypoplasia; echogenic cortices;	NA	NA	NA	Right proptosis; right exotropia
233	CAKUT	CKT-34C	Barua et al. (2014) PMID: 24676634	c.985A>G	p.Thr329Ala	missense	NA	NA	Unilateral	No abnormal	NA	NA	NA	dysmorphic facial syndrome,
234	RCS	8	Okumura et al. (2015) PMID: 26571382	c.1023C>A	p.341Tyr*	nonsense	bilateral optic nerve coloboma	NA	NA	ML, left double ureters	FSGS	NA	CKD3(28y)	NA

Published PAX2 cases were identified from PAX2 LOVD (https://databases.lovd.nl/shared/genes/PAX2, LOVD 3.0 version), Clinvar database (https://www.ncbi.nlm.nih.gov/clinvar/?term=PAX2%5Bgene%5D) and HGMD(http://www.hgmd.cf.ac.uk/ac). Only presumed pathogenic missense variants were retained; if there was doubt as to pathogenicity (e.g. allele inherited from an unaffected parent, or allele listed in ExAC/gnomAD) the case was excluded. Phenotypic data was extracted from original publications. Well-phenotyped affected relatives who were known to be mutation-positive were included, to capture phenotypic variability. For very large pedigrees where not all of the family members could be included, a representative of each phenotype within the family was included.
CAKUT (congenital anomalies of the kidney and urinary trac); CKD (chronic kidney disease); ESRD (end stage renal disease); FSGS (focal segmental glomerulosclerosis); LGD variants (likely/presumed gene disruptive. variants of deletion, frameshift, insertion, truncating and splice site); m, maternal; N.A.(not available); p. paternal; RCS (renal coloboma syndrome); RHD (renal hypodysplasia); UPJO (Ureteropelvic junction obstruction); VUR (vesicoureteral reflux), y, years old.

Supplementary Table S2. Protein structural properties and phenotype predictor values for pathogenic and putatively benign *PAX2* missense variants.

Variants	Phenotype	FoldX (complex)	FoldX (monomer)	SIFT	SIFT_pred	Polyphen2	Polyphen2_HDIV_pred	FATHMM	FATHMM_pred	PROVEAN	PROVEAN_pred	VEST4	MetaSVM	MetaSVM_pred	REVEL	CADD	GERP	phyloP30way_mammalian	LRT	LRT_pred	MutationTaster	MutationAssessor_pred
p.Asp2Gly	non-RCS	0	5.60782	0	D	0.995	D	0.97693	D	-2.48	N	0.719	0.9254	D	0.629	0.92863	0.40864	0.56389	0.13598	N	0.51612	L
p.Met3Leu	Population from gnomAD	N,A	0.354924	1	T	0.21085	B	0.96908	D	-0.38	N	0.57177	0.014	D	0.381	0.44156	0.60666	0.85082	0.38968	N	0.41926	N
p.Met3Val	Population from gnomAD	N,A	0.902478	0.277	D	0.21085	B	0.97006	D	-0.95	N	0.64818	0.2197	D	0.375	0.53352	0.60666	0.85082	0.38968	N	0.42303	N
p.Met3Thr	Population from gnomAD	N,A	0.68241	0.074	D	0.40079	P	0.97343	D	-1.75	N	0.70615	0.5923	D	0.513	0.59096	0.60666	0.52488	0.38968	N	0.44734	N
p.His4Pro	Population from gnomAD	N,A	-0.567788	0.003	D	0.84481	D	0.97583	D	-2.7	D	0.79986	0.6842	D	0.65	0.89392	0.40864	0.85082	0.7519	N	0.53665	M
p.Pro9Ser	Population from gnomAD	N,A	1.10715	0.454	T	0.88582	D	0.97343	D	-2.92	D	0.72209	0.9908	D	0.695	0.80195	0.43689	0.35026	0.00993	U	0.58761	L
p.Pro9Ala	Population from gnomAD	N,A	0.89641	0.029	D	0.86255	D	0.97317	D	-3.01	D	0.71321	1.0036	D	0.696	0.73924	0.43689	0.35026	0.00993	U	0.58761	M
p.Ala12Glu	Population from gnomAD	N,A	-0.297188	0.523	T	0.46291	P	0.97515	D	-2.08	N	0.76666	0.1578	D	0.46	0.60451	0.43689	0.35026	0.20066	U	0.41858	L
p.Gly19Val	Population from gnomAD	1.5498	0.0320908	0.001	D	0.97372	D	0.99508	D	-7	D	0.83167	1.0281	D	0.801	0.87211	0.99707	0.78918	0	D	0.81001	H
p.Gly24Arg	non-RCS	3.3483	5.68048	0	D	1	D	0.99742	D	-6.39	D	0.978	0.9707	D	0.934	0.90557	0.96695	0.78918	0	D	0.81001	H
p.Gly24Glu	non-RCS	3.3483	8.40115	0	D	1	D	0.99734	D	-6.42	D	0.97	0.9779	D	0.908	0.89167	0.99707	0.78918	0	D	0.81001	H
p.Gly25Val	RCS	9.6719	28.0044	0	D	1	D	0.99742	D	-7.16	D	0.99	0.9779	D	0.928	0.88215	0.99707	0.78918	0	D	0.81001	H
p.Gly30Cys	RCS	10.5772	2.64263	0	D	1	D	0.99749	D	-7.43	D	0.972	0.9495	D	0.966	0.91764	0.95888	0.78918	0	D	0.81001	H
p.Gly30Val	RCS	10.5772	2.90733	0	D	1	D	0.99742	D	-7.41	D	0.99	0.983	D	0.972	0.8857	0.95888	0.78918	0	D	0.81001	H
p.Pro32Ser	Population from gnomAD	2.51509	0.627416	0	D	0.92359	D	0.99697	D	-6.46	D	0.96758	0.9625	D	0.956	0.77483	0.95888	0.45946	0	D	0.81001	H
p.Leu33Arg	RCS	3.44679	2.86824	0	D	0.999	D	0.99697	D	-4.89	D	0.984	0.9456	D	0.988	0.90101	0.95888	0.64695	3.1E-05	D	0.81001	H
p.Pro34Ser	Population from gnomAD	1.99595	2.00782	0	D	0.97372	D	0.99697	D	-6.44	D	0.92084	1.0357	D	0.955	0.76113	0.95888	0.45946	0	D	0.81001	M
p.Asp35His	Population from gnomAD	1.93042	0.113424	0.001	D	0.97372	D	0.99464	D	-4.83	D	0.79118	1.0657	D	0.809	0.89335	0.95888	0.78918	0	D	0.81001	M
p.Asp35Asn	Population from gnomAD	1.87563	0.306966	0.039	T	0.92359	D	0.9929	D	-3.58	D	0.47672	1.1354	D	0.65	0.89261	0.95888	0.78918	0	D	0.81001	L
p.Val36Met	Population from gnomAD	-0.554626	-1.30802	0.014	D	0.92359	D	0.99374	D	-2.36	N	0.667	1.1196	D	0.704	0.88257	0.95888	0.78918	0	D	0.81001	N
p.Gln39His	Population from gnomAD	0.750563	-0.415004	0.019	T	0.88582	D	0.99368	D	-3.57	D	0.49965	1.0705	D	0.763	0.74655	0.95888	0.78918	0	D	0.81001	L
p.Ile41Met	Population from gnomAD	1.38238	-0.168786	0	D	0.97372	D	0.99632	D	-2.5	D	0.98563	1.0378	D	0.811	0.67992	0.62375	0.45946	0	D	0.81001	M
p.Ala45Gly	Population from gnomAD	1.33162	0.431612	0	D	0.76916	P	0.99632	D	-3.29	D	0.78737	0.9965	D	0.849	0.81937	0.97798	0.45946	0	D	0.81001	H
p.Gln47Arg	Population from gnomAD	-0.371215	-1.06988	0.024	D	0.78396	P	0.99296	D	-3.09	D	0.71143	1.1179	D	0.733	0.77167	0.97798	0.94714	0	D	0.58761	L
p.Gly48Asp	Population from gnomAD	4.02558	0.873853	0	D	0.97372	D	0.99793	D	-5.78	D	0.92667	0.9532	D	0.843	0.88725	0.97798	0.78918	0	D	0.81001	H
p.Cys52Tyr	RCS	0.190882	-0.273058	0	D	0.999	D	0.99678	D	-9.15	D	0.955	0.9863	D	0.951	0.90327	0.97798	0.78918	0	D	0.81001	H
p.Arg56Gln	non-RCS	0.3383	-0.521884	0.001	D	0.468	D	0.99447	D	-3.33	D	0.842	1.0291	D	0.791	0.76436	0.97798	0.78918	0	D	0.81001	H
p.Ser61Asn	RCS	1.81663	-0.103713	0	D	0.984	D	0.99675	D	-2.58	D	0.953	0.9953	D	0.789	0.77167	0.95557	0.78918	0	D	0.81001	H
p.Ser61Ile	RCS	1.02209	0.638549	0	D	0.999	D	0.997	D	-5.16	D	0.975	0.9619	D	0.895	0.84232	0.95557	0.78918	0	D	0.81001	H
p.Gly63Ser	non-RCS	0.642884	-0.121174	0	D	1	D	0.99727	D	-5.01	D	0.972	1.0348	D	0.893	0.91243	0.98675	0.78918	0	D	0.81001	M
p.Leu69Pro	RCS	5.97708	6.03615	0	D	0.996	D	0.99702	D	-5.98	D	0.979	0.9888	D	0.969	0.91672	0.64147	0.64695	0	D	0.81001	H
p.Arg71Gly	non-RCS	0.713823	1.8169	0	D	0.783	D	0.99666	D	-5.96	D	0.919	0.9649	D	0.979	0.95789	0.98675	0.94714	0	D	0.81001	H
p.Arg71Met	RCS	-1.04788	-0.239569	0	D	0.955	D	0.99672	D	-5.18	D	0.803	0.9671	D	0.922	0.96831	0.98675	0.78918	0	D	0.81001	H
p.Arg71Thr	RCS	-0.170031	0.9144	0	D	0.115	B	0.99658	D	-5.12	D	0.872	1.0166	D	0.804	0.95492	0.98675	0.78918	0	D	0.81001	H
p.Glu74Lys	Population from gnomAD	0.0387142	2.18303	0	D	0.97372	D	0.99592	D	-3.38	D	0.83576	1.0544	D	0.94	0.89891	0.95888	0.78918	1E-06	D	0.81001	M
p.Gly76Ser	RCS	4.17088	4.16124	0	D	1	D	0.99793	D	-5.01	D	0.972	1.0348	D	0.893	0.91243	0.98675	0.78918	0	D	0.81001	H
p.Ser77Asn	Population from gnomAD	0.284257	1.25664	0.002	D	0.82059	D	0.99589	D	-2.57	D	0.75834	1.006	D	0.807	0.7872	0.95888	0.78918	0	D	0.81001	H
p.Pro80Leu	RCS	4.08866	0.412853	0	D	1	D	0.99705	D	-8.66	D	0.966	0.9625	D	0.935	0.80807	0.95888	0.45946	0	D	0.81001	H
p.Ile83Val	Population from gnomAD	0.334122	1.17886	0.004	D	0.70482	B	0.99513	D	-0.84	N	0.55019	1.0582	D	0.734	0.73785	0.95888	0.94714	0	D	0.81001	M
p.Gly85Val	non-RCS	5.79088	8.34901	0	D	1	D	0.99742	D	-7.72	D	0.883	0.9625	D	0.907	0.87162	0.95888	0.78918	0	D	0.81001	H
p.Ala91Val	non-RCS	2.87661	-0.79766	0	D	0.992	D	0.99296	D	-3.49	D	0.506	1.0938	D	0.808	0.80917	0.95888	0.45946	0	D	0.81001	M
p.Thr92Met	non-RCS	-1.45281	0.304363	0	D	1	D	0.99501	D	-5.16	D	0.754	1.0225	D	0.845	0.78092	0.95888	0.45946	0	D	0.81001	M
p.Glu101Gln	Population from gnomAD	-0.339856	0.269271	0.446	T	0.62579	P	0.9946	D	-2.19	N	0.308	1.1169	D	0.603	0.61471	0.95888	0.78918	0	D	0.58761	L
p.Pro107Leu	non-RCS	1.60183	1.08234	0.002	D	0.97372	D	0.99705	D	-8.47	D	0.98268	0.9695	D	0.922	0.81277	0.95888	0.45946	0	D	0.81001	H
p.Pro107Ala	Population from gnomAD	1.90044	5.77055	0.006	D	0.82059	D	0.99691	D	-6.71	D	0.93135	1.0081	D	0.899	0.68943	0.95888	0.45946	0	D	0.81001	M
p.Pro107Leu	Population from gnomAD	1.60183	13.439	0.002	D	0.97372	D	0.99705	D	-8.47	D	0.98268	0.9695	D	0.922	0.81277	0.95888	0.45946	0	D	0.81001	H
p.Ala111Thr	non-RCS	1.26859	0.179639	0	D	0.999	D	0.99468	D	-3.47	D	0.942	1.0294	D	0.869	0.88451	0.95888	0.78918	0	D	0.81001	M
p.Glu113Lys	Population from gnomAD	5.74287	-0.457293	0.001	D	0.975	D	0.99592	D	-3.37	D	0.951	0.96814	D	0.913	4.43336	0.95888	0.78918	0	D	3.91	H
p.Arg115Gln	Population from gnomAD	-0.297793	0.463145	0.001	D	0.88582	D	0.99655	D	-3.39	D	0.97426	1.0055	D	0.916	0.91203	0.67015	0.78918	0	D	0.81001	M
p.Asp116Glu	Population from gnomAD	-0.520878	1.68327	0.05	D	0.76457	P	0.99398	D	-3.33	D	0.82862	1.0639	D	0.857	0.61951	0.67015	0.45946	0	D	0.81001	L
p.Arg117Gln	Population from gnomAD	0.0529551	0.475941	0.002	D	0.84481	D	0.9946	D	-3.32	D	0.64393	1.1061	D	0.867	0.88153	0.95888	0.78918	0	D	0.81001	L
p.Glu121Asp	Population from gnomAD	0.894763	1.54732	0.096	T	0.72444	P	0.99374	D	-2.48	N	0.35194	1.0295	D	0.513	0.44924	0.46726	0.78918	3E-06	D	0.58761	N
p.Gly122Asp	Population from gnomAD	1.68837	1.13509	0.026	D	0.97372	D	0.99508	D	-4.36	D	0.72656	0.4151	D	0.83	0.85043	0.95888	0.78918	0	D	0.81001	M
p.Thr128Ala	Population from gnomAD	0.692799	1.33895	0.056	D	0.46066	B	0.99345	D	-3.78	D	0.49146	1.1041	D	0.694	0.59968	0.95888	0.94714	0	D	0.81001	L
p.Pro130Arg	RCS	14.3728	2.41922	0	D	1	D	0.99705	D	-7.87	D	0.969	0.9695	D	0.973	0.76201	0.95888	0.45946	0	D	0.81001	H
p.Pro130His	RCS	14.3728	2.30038	0	D	1	D	0.99713	D	-7.87	D	0.936	0.9695	D	0.966	0.76274	0.95888	0.45946	0	D	0.81001	H
p.Pro130Ser	non-RCS	14.3728	3.07745	0	D	1	D	0.99697	D	-6.96	D	0.917	0.9895	D	0.974	0.72372	0.95888	0.45946	0	D	0.81001	H
p.Ser131Cys	Population from gnomAD	2.50829	-0.411351	0.001	D	0.92359	D	0.99666	D	-4.14	D	0.86725	0.9845	D	0.914	0.83458	0.95888	0.94714	0	D	0.81001	H
p.Val132Ile	Population from gnomAD	-0.836815	0.00771686	0.002	D	0.51505	B	0.99488	D	-0.86	N	0.69387	1.0957	D	0.63	0.63648	0.95888	0.78918	1E-06	D	0.81001	M
p.Val132Phe	Population from gnomAD	0.446868	-0.259031	0	D	0.78396	D	0.99551	D	-4.32	D	0.88909	0.9846	D	0.918	0.88865	0.95888	0.78918	1E-06	D	0.81001	H
p.Ser133Phe	non-RCS	2.53297	-0.458559	0	D	0.987	D	0.99614	D	-5.36	D	0.947	0.9846	D	0.864	0.76763	0.95888	0.45946	0	D	0.81001	H
p.Ile139Val	non-RCS	0.609232	-0.258986	0.362	T	0.003	B	0.99368	D	-0.72	N	0.577	0.5025	D	0.439	0.40752	0.62125	0.875	0	D	0.58761	N
p.Arg140Gln	RCS	-0.0329847	-0.14249	0.01	D	0.993	D	0.99655	D	-3.31	D	0.978	0.9708	D	0.956	0.80117	0.62125	0.71814	0	D	0.81001	H
p.Arg140Gly	non-RCS	0.799934	0.411429	0.001	D	0.998	D	0.99666	D	-6.02	D	0.958	1.0356	D	0.951	0.74825	0.44525	0.16068	0	D	0.81001	M
p.Arg140Trp	non-RCS	0.318998	-0.289342	0	D	0.999	D	0.99675	D	-6.89	D	0.951	1.0021	D	0.937	0.86254	0.44525	0.16068	0	D	0.81001	H
p.Val143Ile	Population from gnomAD	N,A	-0.274884	0.205	T	0.24676	B	0.9783	D	-0.8	N	0.57433	0.8998	D	0.546	0.59174	0.62125	0.71814	0	D	0.81001	M
p.Gln144His	Population from gnomAD	N,A	0.0135022	0.095	D	0.53321	P	0.98005	D	-3.94	D	0.68082	1.0174	D	0.734	0.68882	0.62125	0.71814	0	D	0.81001	M
p.Pro149Ser	Population from gnomAD	N,A	-0.310827	0.22	T	0.37187	B	0.97772	D	-3.07	D	0.65673	0.7931	D	0.563	0.41235	0.62125	0.38774	0	D	0.81001	L
p.Pro149Thr	Population from gnomAD	N,A	0.536036	0.161	T	0.55432	P	0.97849	D	-2.91	D	0.61087	0.9568	D	0.65	0.63429	0.62125	0.38774	0	D	0.81001	M
p.Thr150Ala	non-RCS	0	-0.0290617	0.756	T	0.002	B	0.97606	D	-1.24	N	0.268	0.5203	D	0.323	0.39669	0.62125	0.875	0	D	0.52396	N
p.Thr150Met	Population from gnomAD	N,A	-0.00454972	0.181	T	0.60758	D	0.9783	D	-1.1	N	0.42149	0.8267	D	0.497	0.59185	0.62125	0.38774	0	D	0.58761	N
p.Thr150Lys	Population from gnomAD	N,A	-0.023833	0.93	T	0.41883	B	0.97628	D	-1.79	N	0.44761	0.8698	D	0.586	0.46804	0.62125	0.38774	0	D	0.58761	L
p.Pro151Leu	Population from gnomAD	N,A	0.213593	0.399	T	0.47102	P	0.97753	D	-2.61	D	0.57518	0.8525	D	0.611	0.56256	0.62125	0.38774	0	D	0.81001	L
p.Pro151Gln	Population from gnomAD	N,A	1.61972	0.62	T	0.57669	P	0.97672	D	-1.01	N	0.56919	0.8935	D	0.518	0.46207	0.62125	0.38774	0	D	0.81001	L

Supplementary Table S2. Protein structural properties and phenotype predictor values for pathogenic and putatively benign PAX2 missense variants.

p.Gly153Arg	Population from gnomAD	N,A	3.58896	0.05 D	0.97372 D	0.9792 D	-2.39 N	0.76573	1.0432 D	0.62	0.9086	0.62125	0.71814	0.00041 D	0.81001 M
p.Ala154Ser	Population from gnomAD	N,A	-0.708549	0.98 T	0.06944 B	0.9783 D	0.12 N	0.16725	-0.1924 T	0.253	0.16952	0.23429	0.24133	0.06739 N	0.24888 N
p.Ala154Thr	Population from gnomAD	N,A	1.03998	0.658 T	0.04355 B	0.97902 D	-0.17 N	0.12627	-0.1659 T	0.256	0.22498	0.23429	0.24133	0.06739 N	0.26065 N
p.Val158Ala	Population from gnomAD	N,A	-0.795897	0.548 T	0.43023 B	0.97693 D	-0.86 N	0.48504	0.7631 D	0.407	0.47207	0.62125	0.5739	0.04963 N	0.53665 M
p.Ala160Thr	Population from gnomAD	N,A	0.586218	0.333 T	0.143 B	0.97812 D	-0.64 N	0.16864	0.2844 D	0.313	0.43581	0.62125	0.71814	6E-06 D	0.37795 N
p.Pro161Ser	Population from gnomAD	N,A	2.00849	0.278 T	0.40572 B	0.9783 D	-3.05 D	0.57263	0.297 D	0.338	0.41245	0.33383	0.38774	0.00037 N	0.51968 L
p.Thr164Asn	non-RCS	0	0.108881	0.233 T	0.92359 P	0.97884 D	-2.21 N	0.82157	1.011 D	0.657	0.57035	0.44666	0.45946	0 D	0.58761 L
p.Thr164Asn	Population from gnomAD	N,A	-0.0666072	0.233 T	0.92359 P	0.97884 D	-2.21 N	0.82157	1.011 D	0.657	0.57035	0.44666	0.45946	0 D	0.58761 L
p.Thr164Ile	Population from gnomAD	N,A	0.468325	0.118 D	0.92359 D	0.97902 D	-3.79 D	0.75742	1.0601 D	0.777	0.74839	0.44666	0.45946	0 N	0.58761 M
p.Val166Ala	Population from gnomAD	N,A	0.762576	0.288 T	0.39298 B	0.97672 D	-2.08 N	0.8266	0.7929 D	0.494	0.53674	0.95557	0.64695	3E-06 D	0.81001 L
p.Ser168Ile	Population from gnomAD	N,A	-1.38881	0.107 D	0.59919 P	0.97989 D	-3.41 D	0.85979	1.0865 D	0.759	0.86254	0.95557	0.78918	0 D	0.81001 M
p.Thr169Arg	Population from gnomAD	N,A	-2.14141	0.483 T	0.67262 D	0.97812 D	-2.54 D	0.83885	1.0617 D	0.701	0.62053	0.66209	0.45946	0 D	0.58761 M
p.Thr169Met	Population from gnomAD	N,A	-2.33932	0.075 D	0.72692 D	0.97902 D	-2.31 N	0.74644	1.0827 D	0.657	0.76912	0.66209	0.45946	0 D	0.58761 M
p.Ala170Thr	Population from gnomAD	N,A	2.19687	0.05 D	0.35598 B	0.97938 D	-2.18 N	0.47115	0.9714 D	0.451	0.48394	0.95557	0.78918	0 D	0.81001 M
p.Ser171Pro	Population from gnomAD	N,A	4.47044	0.365 T	0.79672 D	0.97812 D	-1.82 N	0.80572	1.0511 D	0.657	0.75573	0.95557	0.64695	0 D	0.81001 M
p.Pro173Ser	Population from gnomAD	N,A	0.583109	0.12 T	0.92359 D	0.97956 D	-4 D	0.73465	1.0883 D	0.718	0.66174	0.95557	0.45946	0 D	0.81001 M
p.Val174Ala	Population from gnomAD	N,A	0.201879	0.64 T	0.76457 D	0.97732 D	-1.7 N	0.77601	1.0691 D	0.567	0.48241	0.95557	0.64695	0 D	0.81001 M
p.Ser176Gly	Population from gnomAD	N,A	0.202791	0.164 T	0.75168 D	0.98005 D	-2.73 D	0.72835	1.1009 D	0.746	0.76987	0.95557	0.94714	0 D	0.81001 M
p.Ser176Thr	Population from gnomAD	N,A	-0.1359	0.212 T	0.75168 D	0.9792 D	-1.93 N	0.52475	1.0974 D	0.652	0.41985	0.95557	0.78918	0 D	0.81001 M
p.Ala177Thr	Population from gnomAD	N,A	-0.042309	0.266 T	0.56161 P	0.97866 D	-1.22 N	0.5528	0.9738 D	0.53	0.60666	0.66477	0.78918	0.00192 N	0.81001 M
p.Asn179Ser	Population from gnomAD	N,A	0.069678	0.856 T	0.82059 D	0.9756 D	-0.89 N	0.73735	0.7581 D	0.474	0.51144	0.95557	0.94714	6.2E-05 D	0.81001 L
p.Asn188Ser	Population from gnomAD	N,A	0.397514	0.449 T	0.78936 D	0.97693 D	-2.07 N	0.91621	0.9383 D	0.481	0.5127	0.9274	0.94714	1E-06 D	0.81001 L
p.Gly189Arg	non-RCS	N,A	0.122222	0 D	0.938 D	0.98313 D	-6.11 D	0.993	-0.2534 T	0.924	0.88142	0.9274	0.78918	3E-06 D	0.81001 M
p.Arg195Cys	Population from gnomAD	N,A	0.488423	0.033 D	0.86255 D	0.97902 D	-3.14 D	0.88798	1.0848 D	0.723	0.90954	0.85347	0.45946	0.35294 N	0.81001 M
p.Arg195His	Population from gnomAD	N,A	1.67287	0.367 T	0.86255 D	0.97772 D	-1.38 N	0.87917	1.0032 D	0.599	0.72827	0.85347	0.78918	0.35294 N	0.81001 M
p.Ser196Phe	Population from gnomAD	N,A	6.64772	0.022 D	0.84481 D	0.9802 D	-3.42 D	0.88027	0.7916 D	0.727	0.77348	0.85347	0.45946	0.51049 N	0.81001 M
p.Asn197Ser	Population from gnomAD	N,A	1.02697	0.113 T	0.82059 D	0.97732 D	-1.91 N	0.75192	0.547 D	0.447	0.60114	0.85347	0.94714	0.10377 N	0.58761 M
p.Glu199Lys	Population from gnomAD	0.403	0.78936	-4.09 T	-2 D	0.97732 D	1.0959 N	0.7	0.8776 D	0.7	-0.78292	0.85347	0.78918	0.08857 N	0.81001 M
p.Arg201Lys	Population from gnomAD	0.149	0.78936	-4.09 T	-1.9 P	0.97606 D	1.0318 N	0.598	0.69718 D	0.598	0.0736	0.85347	0.78918	0.02586 N	0.81001 L
p.Arg203Gly	Population from gnomAD	0.11	0.86255	-4.11 D	-4.01 D	0.97866 D	1.0849 D	0.656	0.88089 D	0.656	0.91204	0.85347	0.45946	0.05822 N	0.58761 M
p.Arg203His	Population from gnomAD	0.182	0.88582	-4.14 T	-2.44 D	0.97866 D	-0.7232 N	0.587	0.78643 T	0.587	-0.54302	0.85347	0.78918	0.05822 N	0.81001 L
p.Val206Phe	Population from gnomAD	0.382	0.17295	-4.51 D	-1.04 .	0.97672 D	-0.4511 N	0.349	0.96055 T	0.349	-1.17206	0.80675	0.78918	0.01835 N	0.81001 .
p.Val208Leu	Population from gnomAD	0.844	0.04355	-4.38 T	-0.73 .	0.97369 D	0.0255 N	0.306	0.25259 D	0.306	-1.16576	0.78313	0.78918	0.16194 N	0.81001 .
p.Tyr209His	Population from gnomAD	0.49	0.5227	-4.46 T	-0.52 .	0.9756 D	0.2661 N	0.466	0.268 D	0.466	2.62683	0.78313	0.64695	0.31091 N	0.81001 .
p.Thr210Ala	Population from gnomAD	0.539	0.1009	-4.36 T	-1.19 .	0.97317 D	-0.1386 N	0.474	0.24358 T	0.474	-0.24311	0.78313	0.94714	0.37568 N	0.81001 .
p.Ala213Val	Population from gnomAD	0.276	0.01387	-4.39 T	-0.17 .	0.97394 D	-0.0467 N	0.361	0.24035 T	0.361	1.15572	0.39892	0.16866	0.0686 N	0.81001 .
p.Ile215Val	Population from gnomAD	0.283	0.12992	-4.02 T	-0.09 B	0.96402 D	-0.136 N	0.362	0.24655 T	0.362	0.95596	0.78313	0.94714	0.09636 N	0.81001 N
p.Gly218Arg	Population from gnomAD	0.378	0.78936	-4.06 T	-0.89 D	0.96529 D	0.9307 N	0.535	0.24843 D	0.535	3.15198	0.78313	0.78918	0.00243 N	0.81001 N
p.Arg228Gly	Population from gnomAD	0.289	0.1009	-4.1 T	-0.72 B	0.96659 D	0.0713 N	0.449	0.28783 D	0.449	0.93962	0.78313	0.94714	0.00139 N	0.81001 N
p.Asp229Gly	Population from gnomAD	0.368	0.54061	-4.18 T	-1.4 B	0.97902 D	-0.9271 N	0.625	0.50737 T	0.625	1.32781	0.64645	0.94714	0.00258 N	0.58761 L
p.Pro236Ser	Population from gnomAD	0.321	0.50435	-3.95 T	-3.73 P	0.9756 D	0.8069 D	0.636	0.52404 D	0.636	2.356	0.67838	0.45946	0.00062 D	0.81001 M
p.Pro236Leu	Population from gnomAD	0.098	0.56828	-4.11 D	-4.26 D	0.97812 D	-0.6136 D	0.635	0.58807 T	0.635	1.7736	0.67838	0.45946	0.00062 D	0.81001 L
p.Asn237Ser	Population from gnomAD	0.227	0.24114	-4.16 T	-2.6 B	0.97628 D	0.6164 D	0.313	0.37212 D	0.313	1.01839	0.67838	0.94714	0.41156 N	0.47961 L
p.Asp239Glu	Population from gnomAD	0.207	0.26451	-4.45 T	-2.09 B	0.97884 D	-0.953 N	0.535	0.27893 T	0.535	-0.52946	0.2659	0.0867	0 N	0.4447 M
p.Ser242Asn	Population from gnomAD	0.536	0.46637	-4.15 T	-1.4 P	0.97938 D	-0.5484 N	0.466	0.58046 T	0.466	-1.85023	0.67838	0.78918	0 D	0.45339 L
p.Ser242Thr	Population from gnomAD	0.572	0.49095	-4.16 T	-1.6 P	0.97956 D	-0.6246 N	0.535	0.57925 T	0.535	0.78089	0.67838	0.78918	0 N	0.4486 L
p.Asp245Asn	Population from gnomAD	0.398	0.46994	-4.15 T	-2.29 P	0.9792 D	-0.6197 N	0.556	0.68087 T	0.556	0.44558	0.67838	0.78918	3E-06 D	0.488 M
p.Asp245Glu	Population from gnomAD	0.864	0.15521	-4.04 T	-1.52 B	0.97753 D	-0.9747 N	0.384	0.28226 T	0.384	0.00852	0.48138	0.45946	3E-06 D	0.3297 N
p.Arg248Gln	Population from gnomAD	0.057	0.84481	-4.96 D	-3.09 D	0.98448 D	1.1093 D	0.917	0.91121 D	0.917	0.64525	0.67838	0.78918	1E-06 D	0.81001 M
p.Arg252Gln	Population from gnomAD	0.091	0.84481	-4.77 T	-2.97 D	0.98611 D	1.0989 D	0.843	0.78291 D	0.843	0.48597	0.67838	0.78918	1.3E-05 D	0.58761 M
p.Arg252Leu	Population from gnomAD	0.004	0.86255	-4.86 D	-5.45 D	0.98748 D	1.081 D	0.953	0.9155 D	0.953	0.30638	0.67838	0.78918	1.3E-05 D	0.58761 M
p.Ala253Val	Population from gnomAD	0.228	0.51908	-4.09 T	-2.05 P	0.9783 D	0.9753 N	0.736	0.582 D	0.736	0.83052	0.67838	0.45946	3.9E-05 D	0.52396 M
p.Leu261Pro	Population from gnomAD	0.005	0.97372	-4.67 D	-5.06 D	0.98507 D	0.2909 D	0.956	0.89335 D	0.956	1.04661	0.67838	0.64695	0 D	0.81001 M
p.Arg266Trp	Population from gnomAD	0.009	0.92359	-4.29 D	-4.17 D	0.98035 D	1.0923 D	0.908	0.90585 D	0.908	0.97025	0.47821	0.45946	1.1E-05 D	0.58761 M
p.Arg266Pro	Population from gnomAD	0.13	0.92359	-4.27 D	-3.73 D	0.98005 D	1.0962 D	0.884	0.90777 D	0.884	1.52484	0.67838	0.78918	1.1E-05 D	0.81001 M
p.Arg266Leu	Population from gnomAD	0.09	0.86255	-4.25 T	-3.31 D	0.97973 D	1.0923 D	0.863	0.8974 D	0.863	1.01809	0.67838	0.78918	1.1E-05 D	0.81001 M
p.Arg266Gln	Population from gnomAD	0.265	0.84481	-4.2 T	-1.95 D	0.97866 D	1.0534 N	0.775	0.76157 D	0.775	0.37145	0.67838	0.78918	1.1E-05 D	0.58761 M
p.Arg270Cys	Population from gnomAD	0	0.92359	-5.1 D	-6.33 D	0.987 D	1.0856 D	0.921	0.8892 D	0.921	0.69303	0.67838	0.45946	1E-06 D	0.81001 M
p.Arg270His	Population from gnomAD	0.014	0.92359	-5.06 D	-3.92 D	0.98629 D	0.2617 D	0.942	0.89703 D	0.942	1.02325	0.67838	0.78918	1E-06 D	0.81001 M
p.Pro274Leu	Population from gnomAD	0.001	0.69447	-5.21 D	-6.45 D	0.99066 D	1.0705 D	0.964	0.7514 D	0.964	0.92263	0.67838	0.45946	0 D	0.81001 M
p.Pro274His	Population from gnomAD	0.001	0.76916	-5.22 D	-5.55 D	0.99084 D	1.0663 D	0.955	0.72908 D	0.955	1.78654	0.67838	0.45946	0 D	0.81001 M
p.Val276Ile	Population from gnomAD	0.374	0.82059	-4.2 T	-0.51 D	0.98096 D	0.9959 N	0.638	0.5913 D	0.638	0.32874	0.67838	0.78918	0 D	0.58761 L
p.Ala279Val	Population from gnomAD	0.067	0.38393	-4.08 T	-1.56 B	0.97812 D	-0.8215 N	0.504	0.5832 T	0.504	0.04784	0.67838	0.45946	0 D	0.37677 L
p.Ser280Leu	Population from gnomAD	0.192	0.37346	-4.1 D	-3.47 B	0.97866 D	0.9343 D	0.735	0.63821 D	0.735	-0.86595	0.67838	0.45946	0.00015 D	0.54805 M
p.Glu290Gly	Population from gnomAD	0.257	0.68658	-4.3 T	-3.65 D	0.97973 D	1.0653 D	0.704	0.76614 D	0.704	0.20027	0.77615	0.94714	0.07028 N	0.81001 M
p.Tyr291Cys	Population from gnomAD	0.052	0.76113	-4.19 D	-5.91 D	0.9814 D	1.0704 D	0.723	0.86571 D	0.723	0.89194	0.77615	0.94714	0.32437 N	0.81001 M
p.Tyr291Phe	Population from gnomAD	0.239	0.56292	-4.16 T	-2.64 D	0.98035 D	1.0128 D	0.556	0.58123 D	0.556	0.05132	0.77615	0.94714	0.32437 N	0.58761 M
p.Ser292Ala	Population from gnomAD	0.246	0.4143	-4.11 T	-1.14 P	0.97866 D	0.803 N	0.561	0.44817 D	0.561	2.13139	0.77615	0.64695	0 D	0.44734 M
p.Pro294Ser	Population from gnomAD	0.315	0.58006	-4.02 T	-3.23 D	0.97628 D	0.9538 D	0.668	0.42083 D	0.668	0.4759	0.77615	0.45946	2E-06 N	0.81001 L
p.Ala295Val	Population from gnomAD	0.163	0.184	-4.01 T	-1.75 P	0.97772 D	0.8211 N	0.544	0.39934 D	0.544		0.77615	0.45946	0.04927 N	0.46462 M
p.Ala295Thr	Population from gnomAD	0.428	0.23986	-4.1 T	-1.43 B	0.97792 D	0.572 N	0.329	0.41432 D	0.329	1.81675	0.53973	0.78918	0.04927 N	0.43214 L
p.Leu296Pro	Population from gnomAD	0.046	0.999	-4.37 D	-4.16 D	0.98824 D	1.0431 D	0.811	0.85598 D	0.811		0.77615	0.64695	0.54806 N	0.81001 M
p.Thr297Asn	Population from gnomAD	0.464	0.12133	-4.03 T	-0.56 B	0.97606 D	0.237 N	0.303	0.26648 D	0.303	-0.28889	0.77615	0.45946	0.12364 N	0.44098 N
p.Thr297Ile	Population from gnomAD	0.041	0.44399	-4.1 D	-2.78 P	0.97902 D	0.84 D	0.641	0.50187 D	0.641	1.63056	0.77615	0.45946	0.12364 N	0.44189 L
p.Pro298Ser	Population from gnomAD	0.437	0.02	-3.94 T	-2.24 B	0.97444 D	0.2602 N	0.319	0.28555 D	0.319		0.77615	0.45946	0.01062 N	0.48019 N
p.Gly299Arg	Population from gnomAD	0.053	0.6735	-3.99 D	-4 D	0.97849 D	1.0745 D	0.746	0.81889 D	0.746	-1.89621	0.77615	0.78918	0.01035 N	0.52396 M
p.Leu300Phe	Population from gnomAD	0.25	0.92359	-4.23 D	-2.75 D	0.98487 D	1.1135 D	0.686	0.72465 D	0.686	1.2379	0.77615	0.45946	0.12856 N	0.81001 M
p.Asp301His	Population from gnomAD	0.045	0.77976	-4.19 D	-4.52 D	0.98279 D	1.1128 D	0.708	0.82015 D	0.708	-0.01702	0.77615	0.78918	0.06501 N	0.81001 M
p.Asp301Asn	Population from gnomAD	0.141	0.68407	-4.15 D	-3.28 D	0.98183 D	1.1111 D	0.618	0.88957 D	0.618	0.08974	0.77615	0.78918	0.06501 N	0.81001 M

Supplementary Table S2. Protein structural properties and phenotype predictor values for pathogenic and putatively benign PAX2 missense variants.

p.Asp301Gly	Population from gnomAD	0.238	0.68407	-4.18	D	-4.76	D	0.98245	D	1.091	D	0.69	0.85344	D	0.69	0.11029	0.77615	0.94714	0.06501	N	0.81001	M
p.Lys304Arg	Population from gnomAD	0.298	0.49095	-4.08	D	-2.23	P	0.98183	D	1.0817	N	0.676	0.64524	D	0.676	0.07431	0.77615	0.94714	0.004	N	0.58761	M
p.Ser305Pro	Population from gnomAD	0.335	0.12133	1.47	T	-1.09	B	0.9783	T	0.5344	N	0.067	0.41945	D	0.067	1.93974	0.77615	0.64695	0.35513	N	0.44615	N
p.Ser305Leu	Population from gnomAD	0.134	0.34106	1.53	T	-3.13	B	0.9783	T	0.8985	D	0.114	0.4871	D	0.114	0.05951	0.77615	0.45946	0.35513	N	0.51042	M
p.Thr311Ala	Population from gnomAD	0.436	0.06944	1.67	T	-0.85	B	0.97693	T	-0.0581	N	0.112	0.17954	T	0.112	1.12112	0.09091	0.28842	0.04647	N	0.19599	N
p.Asn312Lys	Population from gnomAD	0.9	0.34953	1.6	D	-2.93	B	0.97693	T	0.626	D	0.041	0.45899	D	0.041	-0.78627	0.53973	0.45946	0.0002	D	0.44406	M
p.Glu314Asp	Population from gnomAD	0.88	0.13708	1.54	T	0.1	B	0.97732	T	0.0018	N	0.079	0.24336	D	0.079	0.17586	0.47038	0.78918	0.00047	D	0.31044	N
p.Leu315Met	Population from gnomAD	0.182	0.60472	1.46	T	-0.9	P	0.98198	T	0.8432	N	0.113	0.6493	D	0.113	0.21395	0.67838	0.45946	0.98866	N	0.53665	M
p.Gly316Asp	Population from gnomAD	0.087	0.59784	1.31	D	-4.01	D	0.98371	T	1.1034	D	0.227	0.68242	D	0.227	0.45218	0.67838	0.78918	0.00012	D	0.54805	M
p.Val319Met	Population from gnomAD	0.109	0.631	1.46	D	-1.69	B	0.98263	T	1.1018	N	0.251	0.72895	D	0.251	-0.98978	0.67838	0.78918	0.00248	N	0.81001	M
p.Pro326Ala	Population from gnomAD	0.087	0.58888	1.61	T	-4.82	P	0.97732	T	1.0119	D	0.106	0.29177	D	0.106	0.61173	0.70563	0.45946	7.7E-05	D	0.54805	M
p.Val327Phe	Population from gnomAD	0.142	0.50647	1.5	D	-3.02	B	0.98183	T	1.0764	D	0.375	0.67694	D	0.375	-0.26436	0.70563	0.78918	0.00001	D	0.81001	M
p.Val327Ile	Population from gnomAD	0.458	0.38116	1.63	T	-0.33	B	0.97884	T	0.6601	N	0.204	0.36696	D	0.204	-0.4513	0.70563	0.78918	0.00001	D	0.58761	L
p.Val327Leu	Population from gnomAD	0.501	0.18783	1.78	T	-1.36	B	0.97753	T	0.3274	N	0.205	0.34723	D	0.205	-0.70899	0.70563	0.78918	0.00001	N	0.81001	L
p.Val327Ala	Population from gnomAD	0.233	0.4371	1.5	T	-2.61	B	0.9814	T	0.9091	D	0.33	0.42398	D	0.33	0.15303	0.70563	0.64695	0.00001	D	0.58761	L
p.Thr329Ala	Population from gnomAD	0.226	0.012	1.65	T	-1.9	B	0.97713	T	0.8635	N	0.101	0.31981	D	0.101		0.47196	0.94714	8.8E-05	N	0.54805	N
p.Thr329Ile	Population from gnomAD	0.038	0.82059	1.49	D	-3.3	P	0.98005	T	1.0967	D	0.241	0.4224	D	0.241	0.38997	0.73479	0.45946	8.8E-05	D	0.58761	L
p.Arg331Cys	Population from gnomAD	0.098	0.7382	0.64	D	-5.22	D	0.52867	T	-0.401	D	0.352	0.96209	T	0.352	-0.0186	0.46572	0.45946	0.02428	N	0.81001	M
p.Arg331His	Population from gnomAD	0.38	0.7382	0.65	D	-2.98	D	0.52642	T	-0.5821	D	0.324	0.94024	T	0.324	0.31556	0.46572	0.78918	0.02428	N	0.52935	M
p.Ala334Val	Population from gnomAD	0.217	0.47443	1.47	T	-1.88	B	0.31987	T	-0.9477	N	0.176	0.64535	T	0.176	0.60409	0.46572	0.45946	0.50399	N	0.34656	L
p.Thr336Ile	Population from gnomAD	0.129	0.86255	1.27	D	-3.63	D	0.36146	T	-0.6693	D	0.385	0.77498	T	0.385	0.79116	0.49832	0.45946	0.0929	N	0.48408	M
p.Thr337Ile	Population from gnomAD	0.134	0.49608	1.27	T	-4.04	P	0.36146	T	-0.6024	D	0.332	0.54404	T	0.332	-0.06204	0.49832	0.45946	0.24333	N	0.58761	M
p.Thr337Asn	Population from gnomAD	0.074	0.6583	1.22	D	-3.25	D	0.37052	T	-0.5522	D	0.256	0.66667	T	0.256	-0.04441	0.49832	0.45946	0.24333	N	0.58761	M
p.Pro343Leu	Population from gnomAD	0.007	0.97372	0.36	D	-7.58	D	0.5788	T	-0.1604	D	0.518	0.63913	T	0.518	1.78623	0.49832	0.45946	0	D	0.81001	M
p.Pro343His	Population from gnomAD	0.002	0.97372	0.34	T	-7.06	D	0.58176	T	-0.2676	D	0.447	0.7412	T	0.447	2.75573	0.49832	0.45946	0	D	0.58761	M
p.Pro343Arg	Population from gnomAD	0.003	0.97372	0.35	D	-7.04	D	0.58029	T	-0.1604	D	0.531	0.74655	T	0.531	2.17627	0.49832	0.45946	0	D	0.58761	M
p.Val345Met	Population from gnomAD	0.198	0.92359	1.04	D	-1.99	D	0.40218	T	-0.4742	N	0.305	0.86321	T	0.305	-0.18597	0.49832	0.78918	0.00005	D	0.46406	M
p.Val345Ala	Population from gnomAD	0.196	0.84481	1.05	T	-2.76	D	0.3999	T	-0.7679	D	0.266	0.81575	T	0.266	0.60487	0.49832	0.64695	0.00005	D	0.43951	M
p.Pro347Ser	Population from gnomAD	0.149	0.86255	1.44	T	-4.25	P	0.57575	T	-0.9077	D	0.218	0.56981	T	0.218	0.36831	0.49832	0.45946	0	D	0.58761	L
p.Pro347Leu	Population from gnomAD	0.04	0.88582	1.33	D	-6.13	D	0.5788	T	-0.6637	D	0.248	0.71296	T	0.248	-0.19584	0.37579	0.45946	0	D	0.81001	M
p.Pro347His	Population from gnomAD	0.004	0.92359	1.3	D	-5.56	D	0.58176	T	-0.6711	D	0.269	0.6853	T	0.269	0.30565	0.37579	0.45946	0	D	0.54805	M
p.Thr348Ser	Population from gnomAD	0.336	0.78936	1.43	T	-1.81	P	0.32958	T	-1.0416	N	0.128	0.52811	T	0.128	-1.15705	0.49832	0.45946	3.8E-05	D	0.43677	L
p.Thr348Ile	Population from gnomAD	0.228	0.88582	1.27	D	-4.03	D	0.36146	T	-0.7228	D	0.293	0.72936	T	0.293	0.19785	0.49832	0.45946	3.8E-05	D	0.48635	M
p.Gly349Ser	Population from gnomAD	0.18	0.92359	1.16	D	-3.72	D	0.38073	T	-0.4793	D	0.399	0.89384	T	0.399	-0.99253	0.49832	0.78918	0.00043	D	0.52935	M
p.Ala359Ser	Population from gnomAD	0.618	0.49095	1.19	T	-1.51	P	0.37578	T	-0.9384	N	0.312	0.53562	T	0.312	0.42872	0.4735	0.78918	5E-06	D	0.58761	M
p.Val362Met	Population from gnomAD	0.091	0.92359	0.78	D	-2.26	D	0.49358	T	-0.2979	N	0.311	0.87391	T	0.311	-0.49827	0.4735	0.78918	6.7E-05	D	0.53665	M
p.Pro363Ser	Population from gnomAD	0.116	0.97372	1.46	T	-4.31	D	0.32238	T	-0.8252	D	0.345	0.76927	T	0.345	0.71354	0.4735	0.45946	1.1E-05	D	0.58761	L
p.Pro363Arg	Population from gnomAD	0.577	0.01387	1.33	D	-3.98	B	0.35031	T	-0.1225	D	0.283	0.09962	T	0.283	0.18225	0.04975	0.17604	0	D	0.08975	.
p.Ser365Arg	Population from gnomAD	0.035	0.60272	1.29	D	-2.71	B	0.35775	T	-1.0033	D	0.175	0.4986	T	0.175	-1.36681	0.13234	0.32254		.	0.38444	.
p.Glu366Asp	Population from gnomAD	0.824	0.16862	1.64	T	-0.81	B	0.27822	T	-0.3581	N	0.062	0.34733	T	0.062	0.65742	0.46726	0.36421		.	0.31015	.
p.Glu366Asp	Population from gnomAD	0.824	0.16862	1.64	T	-0.81	B	0.27822	T	-0.3581	N	0.062	0.34733	T	0.062	0.65742	0.46726	0.36421		.	0.31015	.
p.Gly369Val	Population from gnomAD	0.322	0.06944	N.A.	N.A.	N.A.	B	N.A.	N.A.	-0.1321	N.A.	0.272	0.50149	T	0.272	0.11834	0.15359	0.36421	0.00011	D	0.81001	.
p.Gly369Cys	Population from gnomAD	0.012	0.97372	0.22	D	-5.9	D	0.96627	T	-0.1876	D	0.589	0.19597	T	0.589	0.15376	0.46726	0.36421		.	0.81001	.
p.Gly369Ser	Population from gnomAD	0.1	0.92359	0.28	T	-3.57	P	0.96208	T	-0.2983	D	0.439	0.19743	T	0.439	0.19075	0.46726	0.36421		.	0.81001	.
p.Asn370Ile	Population from gnomAD	0.027	0.88582	1.35	D	-4.38	D	0.34648	T	-0.018	D	0.315	0.48097	T	0.315	-0.33803	0.34374	0.54891		.	0.45182	.
p.Pro371Ser	Population from gnomAD	0.135	0.86255	1.47	T	-3.7	D	0.31987	T	-0.7388	D	0.218	0.18643	T	0.218	1.17967	0.48814	0.32254		.	0.81001	.
p.Pro375Thr	Population from gnomAD	0.084	0.88582	1.37	T	-3.81	B	0.34253	T	-0.8258	D	0.332	0.17605	T	0.332	2.00191	0.48814	0.32254		.	0.81001	.
p.Gln376Pro	Population from gnomAD	0.334	0.75793	1.41	T	-2.54	B	0.33412	T	0.8714	D	0.448	0.74315	D	0.448	-0.25729	0.48814	0.54891		.	0.52935	.
p.Tyr377Asp	Population from gnomAD	0.005	0.88582	0.62	D	-6.62	B	0.53302	T	-0.503	D	0.596	0.74273	T	0.596	0.61821	0.48814	0.35101		.	0.52396	.
p.Thr378Ala	Population from gnomAD	0.179	0.78936	1.47	D	-2.2	B	0.31987	T	-0.9707	N	0.273	0.20557	T	0.273	0.64898	0.33773	0.54891	0.00036	U	0.81001	.
p.Thr378Lys	Population from gnomAD	0.112	0.86255	1.33	D	-2.93	B	0.35031	T	-0.5489	D	0.317	0.19597	T	0.317	-0.15376	0.48814	0.32254		.	0.81001	.
p.Glu382Gln	Population from gnomAD	0.07	0.92359	1.1	D	-1.98	D	0.3905	T	-0.4503	N	0.293	0.18853	T	0.293	0.32087	0.48814	0.36421		.	0.81001	.
p.Ser387Asn	Population from gnomAD	0.273	0.56206	1.39	D	-1.53	P	0.33842	T	-0.9469	N	0.154	0.78612	T	0.154	-0.27824	0.36363	0.3622		.	0.36999	.
p.Pro389His	Population from gnomAD	0.001	0.88582	1.29	D	-4.51	D	0.35775	T	0.7366	D	0.455	0.48987	D	0.455	1.09078	0.48814	0.32132	0.05323	U	0.81001	.
p.Ala390Pro	Population from gnomAD	0.21	0.59664	1.3	D	-2.36	D	0.3559	T	-0.7353	N	0.376	0.2074	T	0.376	4.15411	0.48814	0.3622		.	0.81001	.
p.Pro395Ala	Population from gnomAD	0.043	0.78396	1.42	D	-4.33	D	0.33189	T	-0.6676	D	0.348	0.34127	T	0.348	0.81055	0.42236	0.32767		.	0.81001	M
p.Pro395Arg	Population from gnomAD	0.012	0.86255	1.41	D	-4.85	D	0.33412	T	-0.6137	D	0.539	0.34165	T	0.539	0.28031	0.42236	0.32767		.	0.81001	M
p.Ser399Asn	Population from gnomAD	0.057	0.68788	1.21	D	-1.8	D	0.3723	T	-0.5446	N	0.227	0.36783	T	0.227	1.3181	0.42236	0.38364		.	0.81001	M
p.Ala401Val	Population from gnomAD	0.211	0.23361	1.38	D	-1.22	B	0.3405	T	-1.0419	N	0.081	0.35631	T	0.081	-0.7003	0.42236	0.32767		.	0.81001	.
p.Pro407Ala	Population from gnomAD	0.176	0.78396	1.52	T	-3.85	D	0.30669	T	-0.8246	D	0.283	0.33355	T	0.283	0.49092	0.42236	0.32767		.	0.81001	.
p.Pro407Arg	Population from gnomAD	0.002	0.86255	1.41	D	-4.71	D	0.33412	T	-0.6485	D	0.424	0.33806	T	0.424	0.04076	0.42236	0.32767		.	0.81001	.
p.Ala408Asp	Population from gnomAD	0.016	0.28043	1.48	D	-0.88	B	0.31731	T	-0.2958	N	0.132	0.32929	T	0.132	0.89459	0.42236	0.32767		.	0.81001	.
p.Ala409Ser	Population from gnomAD	0.17	0.27757	1.57	D	0.06	B	0.29342	T	-1.0567	N	0.061	0.35306	T	0.061	-1.35694	0.3215	0.38364		.	0.81001	.
p.Ala411Thr	Population from gnomAD	0.226	0.78396	0.97	D	-0.79	D	0.42502	T	-0.4639	N	0.272	0.35641	T	0.272	0.97688	0.42236	0.38364		.	0.81001	.
p.Ala413Pro	Population from gnomAD	0.037	0.66185	1.23	D	-1.77	D	0.36872	T	-0.5883	N	0.236	0.35584	T	0.236	6.18975	0.42236	0.38364		.	0.81001	.
p.Ala413Val	Population from gnomAD	0.025	0.60522	1.29	D	-1.69	D	0.35775	T	-0.1878	N	0.141	0.34685	T	0.141	0.79891	0.42236	0.32767		.	0.81001	.
p.Tyr414Phe	Population from gnomAD	0.123	0.72923	1.38	T	-1.78	D	0.3405	T	-0.8871	N	0.23	0.37661	T	0.23	-0.22681	0.42236	0.5753		.	0.81001	.
p.Arg416Leu	Population from gnomAD	0.007	0.75477	1.49	D	-2.32	D	0.3147	T	-0.6665	N	0.264	0.35679	T	0.264	0.92136	0.42236	0.38364		.	0.81001	.
p.His417Tyr	Population from gnomAD	0	0.63021	-4.43	D	-2.16	P	0.97492	D	1.0821	N	0.773	0.34904	D	0.773	-0.81721	0.42236	0.32767		.	0.81001	.
p.His417Pro	Population from gnomAD	0	0.71173	-4.47	D	-3.67	D	0.97583	D	-0.7156	D	0.865	0.37574	T	0.865	1.79241	0.42236	0.5753		.	0.81001	.

Reference

1.Dong C, Wei P, Jian X, Gibbs R, Boerwinkle E, Wang K* and Liu X*. (2015) Comparison and integration of deleteriousness prediction methods for nonsynonymous SNVs in whole exome sequencing studies. Human Molecular Genetics 24(8):2125-2137

2. Liu X, Li C, Mou C, Dong Y, and Tu Y. 2020. dbNSFP v4: a comprehensive database of transcript-specific functional predictions and annotations for human nonsynonymous and splice-site SNVs. Genome Medicine. 12:103
